# Magnetic Solitons in Hierarchical 3D Magnetic Nanoarchitectures of Nanoflower Shape

**DOI:** 10.1021/acs.nanolett.4c04584

**Published:** 2024-11-26

**Authors:** Olha Bezsmertna, Rui Xu, Oleksandr Pylypovskyi, David Raftrey, Andrea Sorrentino, Jose Angel Fernandez-Roldan, Ivan Soldatov, Daniel Wolf, Axel Lubk, Rudolf Schäfer, Peter Fischer, Denys Makarov

**Affiliations:** † Helmholtz-Zentrum Dresden-Rossendorf e.V., Institute of Ion Beam Physics and Materials Research, 01328 Dresden, Germany; ‡ Kyiv Academic University, 03142 Kyiv, Ukraine; ¶ Department of Physics, University of California, Santa Cruz, California 95064, United States; § Materials Sciences Division, Lawrence Berkeley National Laboratory, Berkeley, California 94720, United States; ∥ Alba Light Source, MISTRAL beamline, Cerdanyola del Vallès 08290, Spain; ⊥ Leibniz Institute for Solid State and Materials Research, 01069 Dresden, Germany

**Keywords:** curvilinear nanomagnetism, 3D nanoarchitectures, magnetic solitons, symmetry break

## Abstract

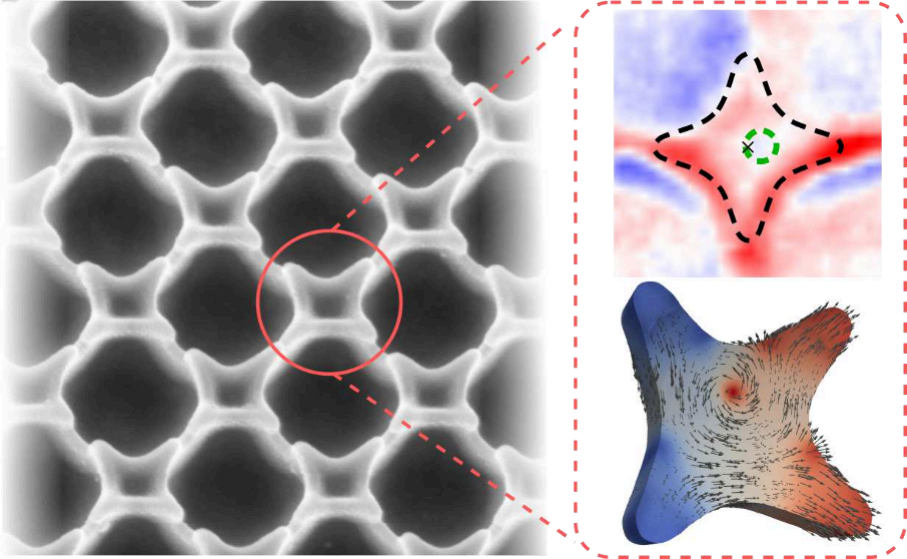

Curvilinear magnetism emerged as a new route to tailor properties of magnetic solitons by the choice of geometry and topology of a magnetic architecture. Here, we develop an anodized aluminum oxide template-based approach to realize hierarchical 3D magnetic nanoarchitectures of nanoflower shape. The technique provides defect-free regular arrays of magnetic nanoflowers of tunable shape with a period of 400 nm over cm^2^ areas. We combined advanced magnetic imaging methods with micromagnetic simulations to study complex magnetic states in nanoflowers originating due to magnetostatics-driven symmetry break in curvilinear nanomembranes. An interaction between surface and volume magnetostatic charges in 3D curved nanoflowers leads to the stabilization of asymmetric and shifted vortices as well as states with two Bloch lines. Ordered large area arrays of complex-shaped magnetic nanoarchitectures developed in this work are relevant for prospective research on 3D magnonics and spintronics.

Curvilinear and three-dimensional (3D) nanomagnetism represents a rapidly advancing area of nanotechnology, requiring novel approaches in design, fabrication, characterization and exploration of the application potential of geometrically curved magnetic nanoarchitectures.^1−3^ These nanoscale objects exhibit various topological and geometric effects that do not reveal themselves in planar (2D) thin films or nanostripes.^4^ 3D magnetic structures offer greater degree of flexibility in the design of magnetic states by exploring the topology of the object’s shape,^5^ enabling space-inversion symmetry break due to geometry and associated Dzyaloshinskii—Moriya interaction (DMI)^6^ and effects related to nonlocal chiral symmetry break^7,8^ to mention just a few appealing fundamental future research directions. Due to their peculiar properties and the ability to geometrically tailor their magnetic states, 3D magnetic nanostructures have the potential to revolutionize a wide range of device applications (from data storage and spintronics to biomedical devices and energy harvesting systems) by enhancing their performance, functionality and efficiency.

Numerous synthesis techniques have already been developed to fabricate 3D magnetic nanostructures. Self-assembly of nonmagnetic nanoparticles capped by magnetic films is an effective method.^9−11^ However, self-assembled nanostructures often contain a large number of defects, significantly reducing the homogeneity of the magnetic properties across the sample. Although two-photon lithography^12,13^ can provide high resolution and enables, e.g. wireframe structures with high aspect ratios, it is primarily suited for creating single nanoobjects because of a moderate processing speed limiting large-scale, high-yield fabrication. 3D printing techniques, such as focused electron/ion beam induced deposition (FEBID and FIBID),^14−16^ enable precise construction of arbitrary-shaped nanostructures, allowing for intricate designs with high spatial resolution.^17^ However, these methods face similar challenges with time efficiency, therefore allowing fabrication of only a limited number of complex-shaped nanoobjects. For many practical applications, such as spintronic and magnonic devices, it is necessary to fabricate tunable 3D magnetic nanostructures with high uniformity over large areas using scaleable synthesis approaches. However, these requirements are beyond the capabilities of currently available techniques. Unlike the above-mentioned methods, the anodized aluminum oxide (AAO) template-based approach enables several significant advantages such as large-scale, continuous nanostructure arrays of high quality. It allows for a high degree of complexity and precise geometrical tunability of the resulting nanostructures.^18^

Here, we present a method to fabricate large areas of highly periodic flower-shaped soft magnetic nanoobjects with a complex 3D profile and provide an analysis of magnetic microstructures in these novel magnetic nanoarchitectures. Characterizing those objects by a suite of complementary imaging techniques, such as magnetic transmission soft X-ray microscopy (MTXM), electron holography, magneto-optical Kerr effect (MOKE) microscopy, and interpreting the data by micromagnetic simulations, we demonstrate that soft magnetic nanoflowers support a variety of topologically nontrivial curvature-stabilized microstructures including asymmetric and shifted vortices as well as exotic states with two Bloch lines, while large valleys between nanoflowers preserve the uniform magnetic state and develop μm-size interaction domains.^19^ We show that the specific property of these 3D geometries has the potential to focus on the study of interaction between the surface and volume magnetostatic charges leading to the highly asymmetric magnetic ground states.

To produce a curvilinear magnetic film with hierarchical ordering of shapes, we use an electropolished aluminum foil as a substrate. A square pattern of nanoindentations with a period of 400 nm is produced by a stamp decorated with a periodic array of nanopillars (Figure 1a). This procedure results in shallow nanoindentations, which are then covered by Al_2_O_3_ during subsequent anodic oxidation. The oxidation process of the stamped nanoindentations is supplemented by the formation of nanocavities between indentations (Figure 1b) due to uneven oxidation rates across the entire substrate surface. The method offers a versatile platform to form different 3D nanostructures with tunable depth and shape, which is controlled by the oxidation time in conjunction with wet chemical etching. In particular, the etching time is an efficient parameter to tune the final topography of the substrate. Larger nanoindentations are characterized by a rounded shape similar to a hemisphere with a square-like deformation. Regions with nanocavities in the center produce flower-shaped structures of the same square symmetry across the entire stamped area(Figure 1c). The petals of nanoflowers can be either connected or separated (Supporting Figure 2) depending on the wet etching time. Finally, we perform magnetron sputter deposition of Fe_20_Ni_80_ alloy (Permalloy) films with a thickness of 30 nm, 40 and 50 nm (Figure 1d). We use 5 nm-thick Ta buffer and capping layers. Scanning electron microscopy (SEM) images of 50 nm-thick Permalloy nanoflowers are shown in Figure 1e,f. Each nanoflower has the smallest and largest lateral extensions: 170 nm (inner diameter of the nanoflower) and 350 nm (outer diameter of the nanoflower that includes petals). The nanoflower depth is about 50 nm. The distance between petals of neighboring nanoflowers is about 40 nm. Valleys between nanoflowers are of 370 nm in diameter and 350 nm in depth (Supporting Figure 3). Due to shadowing effect in a directed deposition, the magnetic film has a constant thickness profile along the deposition direction which results in the modulated thickness profile with respect to the surface normal (Supporting Figure 4).

**Figure 1:**
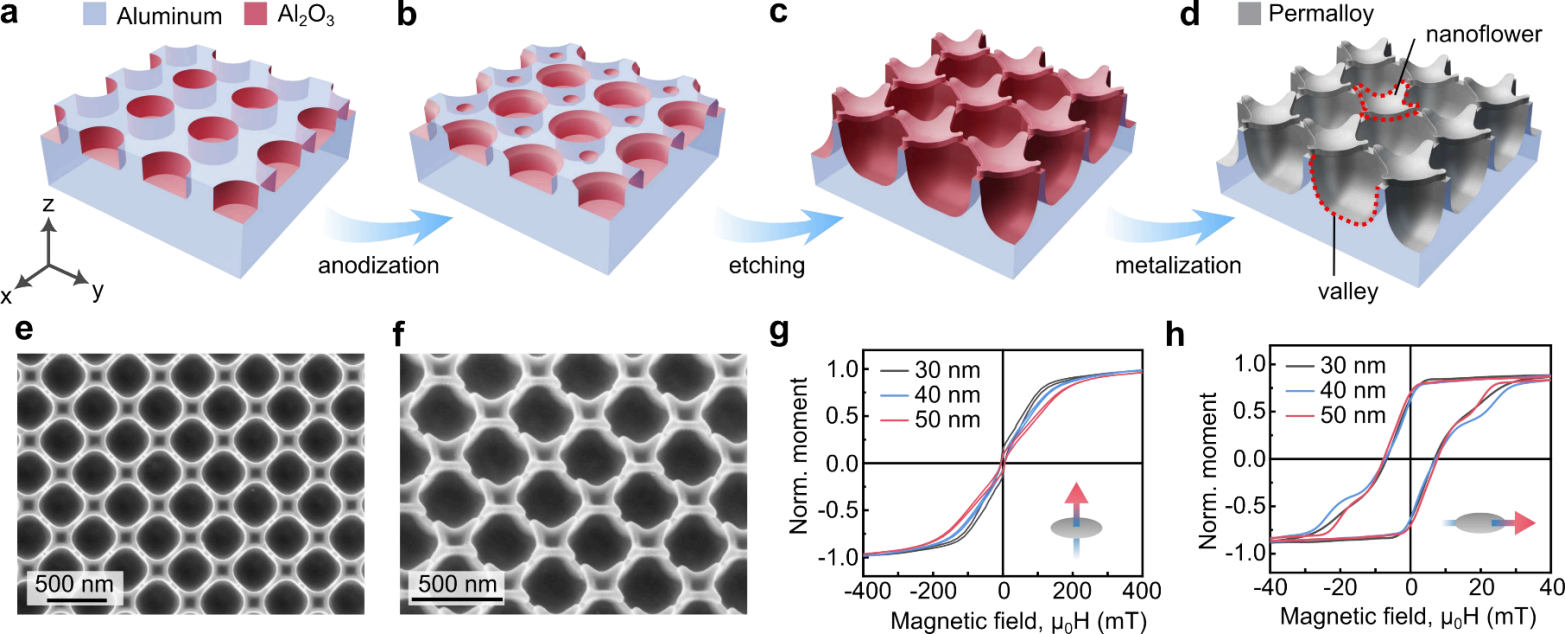
Fabrication and magnetic characterization of arrays of Permalloy nanoflowers. Panels (a-d) show schematics of the process to prepare magnetic nanoflowers. (a) Aluminum foil decorated with nanoindentations is (b) anodized to fine-tune the surface profile and shape of cavities. (c) Wet chemical etching of Al_2_O_3_ nanoporous membranes provides a template with a flower-like surface topography and hemispherical cavities in between. (d) Magnetic nanostructures are prepared by deposition of Permalloy thin films of different thickness on top of the template. Scanning electron microscopy (SEM) reveals (e) the top-view and (f) tilted-view of a 50 nm-thick Permalloy layer on nanoflower templates. Magnetic hysteresis loops measured with magnetic field applied (g) perpendicular and (h) parallel to the sample plane for three different thicknesses of the Permalloy layer.

Integral magnetic properties of the samples are characterized using vibrating sample magnetometry (VSM). Magnetic hysteresis loops measured in the out-of-plane geometry of the applied magnetic field reveal the expected hard-axis shape yet indicate the presence of a small out-of-plane magnetic moment at remanence (Figure 1g). The out-of-plane moment stems from the parts of the Permalloy films deposited on the inclined faces of valleys. The in-plane hysteresis loops (Figure 1h) show the so-called “wasp-waisted” shape.^20−22^ This shape of the loops is an indication either of two independent reversal modes (e.g., flowers vs valleys) or nucleation of magnetic vortices.^23−26^

To understand the magnetic reversal process and its relation to the 3D shape of the magnetic thin film, we perform imaging of magnetic states at nanoscale using transmission soft X-ray and electron microscopies. For these measurements, we develop an approach to realize freestanding Permalloy nanomembranes from the films deposited on alumina templates described above. We coat the samples with a protective layer of poly(methyl methacrylate) (PMMA), see Figure 2a, and etch the bulk Al substrate in a solution of CuCl_2_+HCl (Figure 2b). Then, the Al_2_O_3_ layer under the Permalloy film is etched away in a water solution of NaOH. The protective PMMA layer is removed in an acetone bath (Figure 2c). A freestanding membrane with nanoflower arrays keeps its shape floating on the water surface due to its sufficiently high tension. The resulting nanomembrane is transferred to a transmission electron microscopy (TEM) Cu grid (Figure 2d). SEM images of the top and bottom sides of a transferred nanomembrane demonstrate that individual nanoflowers and valleys in between them maintain their initial geometry without deformations (Figure 2e). Magnetic characterization of the samples before and after transfer using MOKE magnetometry reveals that the main features of the hysteresis loop remain unchanged (Figure 2f). The shape of the hysteresis loop in MOKE after the sample transfer can be different due to small deformations of the transferred membrane, which can also cause local modifications of the strain distribution. The observed lowering of the saturation field might be related to the strain relaxation in the free-standing nanomembrane. Furthermore, MOKE microscopy enables access to the magnetic domain pattern on a larger scale (resolution of about 600 nm). In particular, we observe interaction domains (Figure 2g–j), resembling spikes with 4 predominant directions of growth due to 4-fold geometrical symmetry of the sample structure. Characteristic dimensions of the interaction domains are larger than the geometric unit cell of the template, i.e. 400 nm.

**Figure 2:**
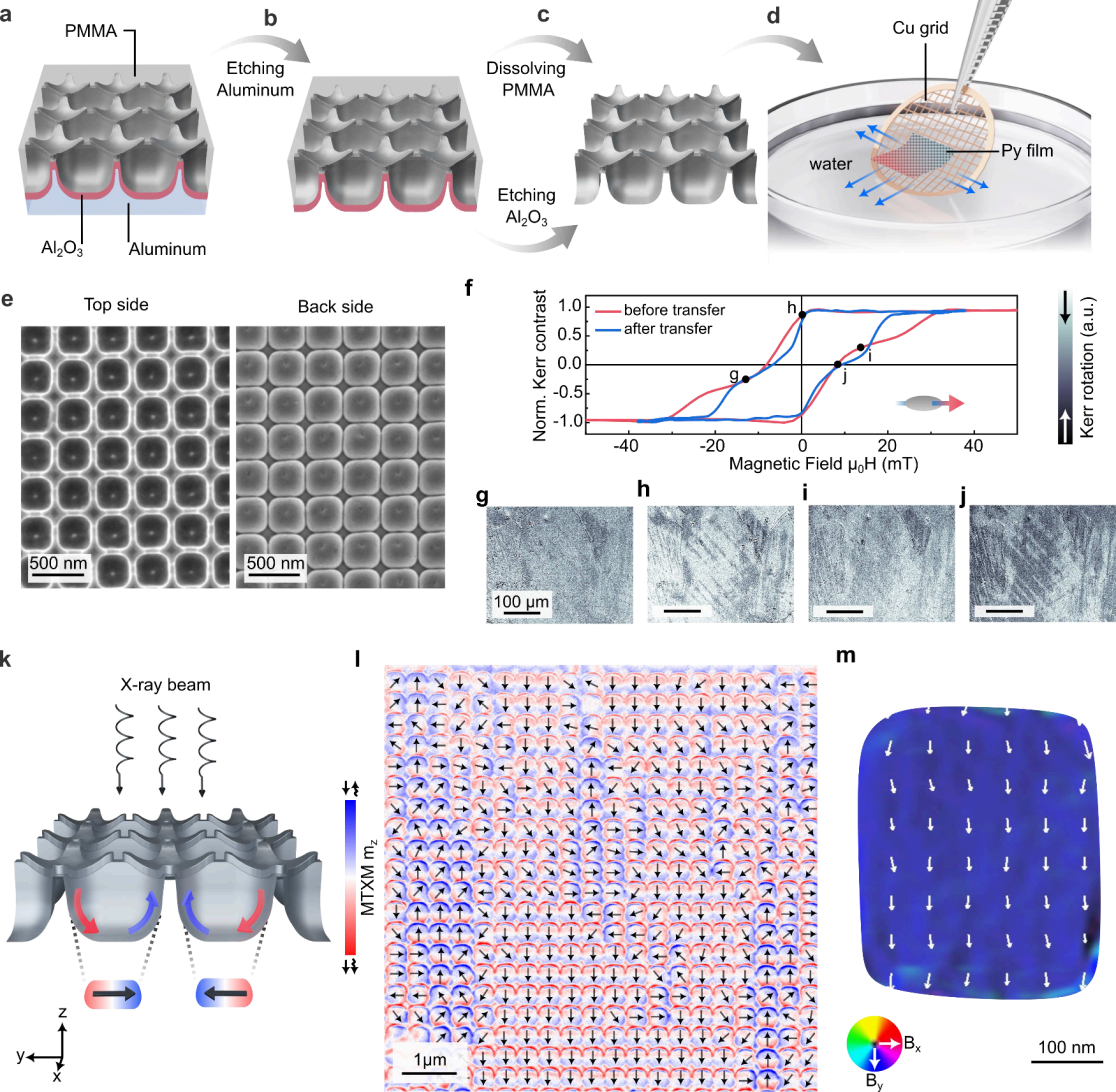
Magnetic states in freestanding nanomembranes of Permalloy 3D nanoarchitectures. Panels (a-d) show schematics of the release of a nanomebrane and its transfer to a TEM Cu grid. (a) A substrate with curvilinear Permalloy film is covered by a polymeric PMMA layer followed by (b) etching of aluminum. (c) A freestanding curvilinear Permalloy nanomembrane is obtained after etching the Al_2_O_3_ layer and dissolving the PMMA layer. The nanomembrane is then immersed in water. (d) A TEM Cu grid is used to fish out a curvilinear Permalloy nanomembrane. (e) SEM images of the top and back sides the freestanding curvilinear Permalloy films (cf. Figure 1e). (f) Magnetic hysteresis loops measured using MOKE magnetometry before and after transfer. (g–j) Exemplary magnetic states imaged using MOKE microscopy revealing the presence of interaction domains. The corresponding points are indicated on the hysteresis loop. (k) Schematic of the transmission MTXM measurement and the origin of the in-plane magnetic contrast in geometrically curved Permalloy films. (i) Large field of view MTXM image of the curvilinear Permalloy nanomembrane with several interaction domains. The magnetic state in valleys is indicated with arrows. (m) Mapping of the in-plane components of the projected magnetic induction (B_*xy*_) inside a single valley reconstructed by off-axis electron holography.

A freestanding curvilinear Permalloy nanomembrane is analyzed using MTXM. Figure 2k shows schematics of the experiment with the normal incidence of the circularly polarized X-rays on the magnetic nanomembrane. In this geometry, the MTXM is sensitive to the out-of-plane magnetization component that can be present on the inclined parts of the sample, e.g. valleys or nanoflowers, domain walls or Bloch lines. An exemplary image of the magnetic state of the sample area spanning over about 380 geometric unit cells is shown in Figure 2l. The magnetization direction determined according to the schematics in Figure 2k is indicated by black arrows. Each valley has an almost uniform magnetic state with the magnetization following its surface. The uniform state of the valleys is further confirmed by high-resolution electron holography measurements (Figure 2m, Supporting Figure 8), revealing the in-plane components of the projected magnetic induction. We note that the experimentally observed magnetic states in valleys are different compared to the case of individual Permalloy spherical cap structures of similar dimensions, which stabilize vortices.^27^ These vortices appear due to a clearly defined edges of magnetic caps. The experimentally observed preference to have the uniform magnetic state in valleys can indicate a different state on the valley edges, which suppresses the vortex state. While the direct interpretation of the magnetic contrast at the boundaries of the valleys in Figure 2l is challenging because of the overlap of the signal between valleys and petals of nanoflowers, the observed uniform magnetization can be a signature of a magnetic connection between neighboring geometric unit cells. The magnetization direction in the valleys mainly follows one of the symmetry axes of a square, which is in agreement with their 4-fold symmetry observed in SEM (Figure 2e).

Based on the MTXM data, we access the statistical distribution of magnetic states of individual nanoflowers. We note that the interpretation of the magnetic state in a particular nanoflower is possible only within it is central region, because the signal from petals is overlapped with the signal from edges of the valleys. A major amount of nanoflowers with the clearly identifiable magnetic state (46%) host a Bloch line shifted from the origin (Figure 3a). 32% of nanoflowers reveal the so-called magnetic flower state with the symmetry axis oriented along the shortest lateral dimension of the nanoflower (Figure 3b). The least observed states correspond to several Bloch lines (Figure 3c, 20%) and to the Bloch line in the center of the flower (Figure 3d, 2%).

**Figure 3:**
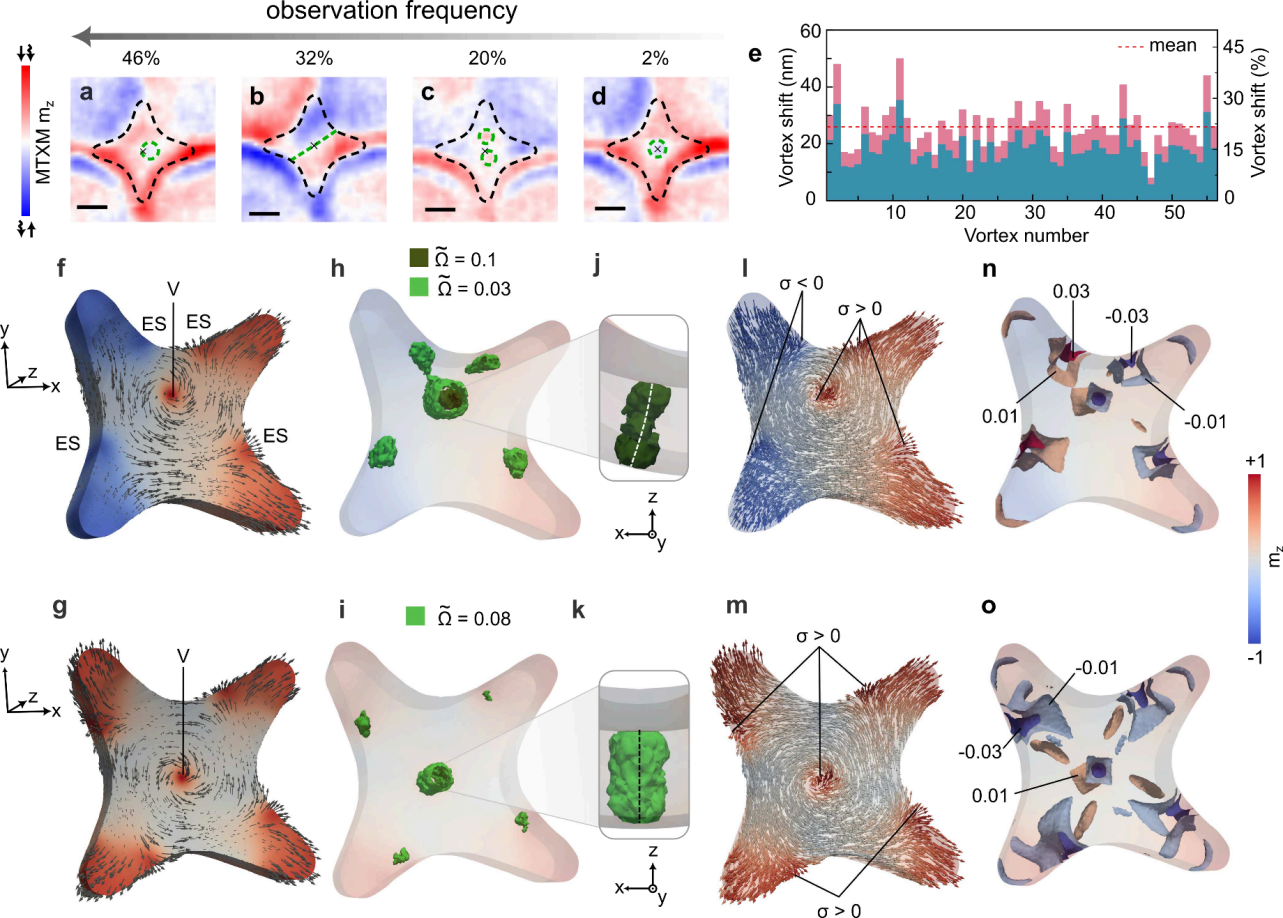
Magnetic states of individual Permalloy nanoflowers. Different magnetic states observed experimentally in individual nanoflowers using MTXM. (a) Magnetic vortex shifted from the center (lowest energy state). (b) A magnetic flower state. (c) Two magnetic vortices within the same nanoflower. (d) Vortex in the center of a nanoflower. In panels (a–d) the scale bar is 100 nm. (e) Mean shift of the vortex core from the geometrical center of nanoflowers measured for 56 vortices like the one shown in (a). Micromagnetic simulations of the lowest-energy shifted vortex (c.f. panel (a)) and higher-energy centered vortex (cf. panel (d)) states are shown in panels (f) and (g), respectively. “V” stands for the vortex core, “ES” stands for the edge state. Distributions of the topological charge flux densities Ω̃, as well as values if isosurfaces for both states are indicated in panel (h) for the shifted vortex and (i) for the centered vortex. Close-up side-view of a Bloch line of the shifted vortex shown in panel (j). For the centered vortex, the close-up is shown in panel (k). The center of Bloch lines is indicated with a dashed line. Surface charges are shown in (l,m). Volume charges are shown in (n,o).

A further interpretation of the observed states, we perform micromagnetic simulations of individual nanoflowers whose geometry is reconstructed based on SEM images (Supporting Figure 3). We found that for a 50 nm-thick Permalloy nanoflower, the ground state corresponds to the vortex whose Bloch line is shifted from the origin. This shift results in the whole magnetic symmetry being characterized by the vertical mirror plane combined with time inversion, *m*′ (Figure 3f). We note that a comparison between simulations (Figure 3f) and MTXM data (Figure 3a–d) can be done only within the central region of the flower, because the MTXM contrast in the petals is overlapped with the underlying side walls of the valleys (c.f. bask side in Figure 2e). At side faces of the nanoflower, there are four edge states represented by half an tivortices (Figure 3f,g, where the presence of the topologically nontrivial states characterized by the topological invariant of the  homotopy group is indicated in terms of the topological charge flux density Ω̃^28,29^). This is in agreement with the analysis of the average vortex shift from the geometric center of a nanoflower by about 25 nm (Figure 3e). The magnetic state is determined by the interaction of surface and volume magnetostatic charges, which is well pronounced in curvilinear geometries.^8^ Namely, the presence of surface and volume charges of opposite sign in the vicinity of each other (top part of the projection shown in Figure 3g) leads to their stronger cross-interaction and lowering of the total magnetostatic energy in comparison with more symmetric states, where the distance between magnetostatic charges of opposite sign is larger. Furthermore, the curvilinear geometry of nanoflowers forces a Bloch line to be bent in the ground state with the average curvature of about 0.027 nm^–1^ (Figure 3h,j). The twist is absent within the numerical accuracy of simulations which is in agreement with a sufficiently high symmetry of the nanoflower geometry. The nucleation of vortices as low-energy states can be also supported by the positive Gaussian curvature of the nanoflower geometry.^30^ Other magnetic states like a magnetic flower state (Figure 3b), several vortices (Supporting Figure 15) or a symmetric vortex in the center of the geometry (Figure 3g,i) have higher energies, which is in agreement with the frequency of their observation in experiment (Supporting Figures 12, 13).

In summary, we developed a method to fabricate highly ordered arrays of magnetic 3D objects of complex shape over areas of several cm^2^. In this work, we focus on objects possessing a nanoflower shape with 4 petals separated by close-to-hemispherical nanoindentations. These objects were capped with soft magnetic Permalloy thin films revealing complex magnetic states at the location of nanoflowers. These states include asymmetric and symmetric vortices, flower-state magnetization distributions and multiple vortices within a single nanoflower, which are observed experimentally and analyzed using micromagnetic simulations. The properties of magnetic states are determined by the geometry-induced interaction between the surface and volume magnetostatic charges. These ordered arrays of magnetic architectures of complex shape can enable further explorations in nonlinear magnetization dynamics (solitons within a nanoflower), 3D magnonics,^31,32^ and curvilinear spintronics.^33^ Furthermore, the possibility to realize highly curved objects can be used to search for curvature-induced skyrmions in out-of-plane magnetized thin films.^34^

We want to note that the fabrication method proposed here is not limited to magnetic materials. We anticipate that these templates can be used for the realization of different families of curvilinear electronic materials,^3,35^ e.g. can act as curvature templates for modifying transport properties in 2D materials.^36^

Our approach of fabrication of 3D magnetic architectures can be readily extended toward fabrication of hierarchical magnetic nanostructures with unconventional geometry-driven properties with excellent structural uniformity over centimeter-long scales and further upscaled to a wafer size with roll-to-roll techniques paving the way for industrial fabrication of practical applications of 3D magnetic nanoarchitectures.

## Supporting Information Available

Further details of sample fabrication, including geometry tailoring, experimental details of (i) magnetic transmission X-ray microscopy (MTXM), (ii) electron holography, and (iii) MOKE microscopy and magnetometry as well as description of micromagnetic simulations (PDF)
