# Supplementary material for: Magnetic Solitons in Hierarchical 3D Magnetic Nanoarchitectures of Nanoflower Shape
Source: Nano Lett. 2024 Nov 26;24(49):15774–80. doi: 10.1021/acs.nanolett.4c04584 (PMC11639047; doi:10.1021/acs.nanolett.4c04584)
Supplement: Supplementary file 1 — nl4c04584_si_001.pdf [file nl4c04584_si_001.pdf]

Supporting information for
"Magnetic solitons in hierarchical 3D magnetic nanoarchitectures of nanoflower shape"

Olha Bezsmertna,[1] Rui Xu,[1,*] Oleksandr V. Pylypovskyi,[1,2,†] David Raftrey,[3,4]
Andrea Sorrentino,[5] Jose Angel Fernandez-Roldan,[1] Ivan Soldatov,[6] Daniel Wolf,[6]
Axel Lubk,[6] Rudolf Schäfer,[6] Peter Fischer,[3,4] and Denys Makarov[1,‡]

[1]*Helmholtz-Zentrum Dresden-Rossendorf e. V., Institute of Ion Beam Physics and Materials Research, 01328 Dresden, Germany*
[2]*Kyiv Academic University, Kyiv 03142, Ukraine*
[3]*Department of Physics, University of California, Santa Cruz, 95064, California, USA*
[4]*Materials Sciences Division, Lawrence Berkeley National Laboratory, Berkeley, 94720, California, USA*
[5]*Alba Light Source, MISTRAL beamline, Cerdanyola del Vallés 08290, Spain*
[6]*Leibniz Institute for Solid State and Materials Research, 01069 Dresden, Germany*

(Dated: November 18, 2024)

CONTENTS

I. Details on magnetic transmission soft X-ray microscopy (MTXM) 2

II. Details on electron holography 3

III. Details on magneto-optical Kerr effect (MOKE) measurements 4

IV. Micromagnetic simulations 5

References 25

* r.xu@hzdr.de
† o.pylypovskyi@hzdr.de
‡ d.makarov@hzdr.de

I. DETAILS ON MAGNETIC TRANSMISSION SOFT X-RAY MICROSCOPY (MTXM)

MTXM images were acquired at the full field Soft X-ray Transmission microscope (TXM) installed at the Mistral beamline of the ALBA Synchrotron [1]. Circular polarized and monochromatic radiation was obtained depleting the electron orbit in the bending magnet source and using a variable line spacing plane grating monochromator (resolving power of about 2000 with exit slit at 15 μm). It was used to illuminate a single bunch glass capillary which focalize the radiation on the sample. After the sample, a Fresnel zone plate with 25 nm outermost zone width was used as objective lens to create a magnified image of the sample (MAG = 1450, effective pixel size $\approx 9\,\mathrm{nm}$) on the charge coupled device (CCD) detector (Pixis XO 1024, Oxford Instrument). The corresponding field of view was about 8.9 μmx 8.9 μm. High vacuum condition was kept during all the measurements.

The Fe L_3 absorption edge energy was selected to observe magnetic contrast from the permalloy sample. After region of interest selection and focus optimization, for each polarization a repetition of 60 images, 2 seconds exposure time each one, was acquired together with the flat field (FF, i.e. the incident intensity). The flat field image was recorded removing completely the sample from the X-ray beam. In this case 30 images, instead of 60, were collected. A proper processing of these raw DATA is crucial for the quality of the final result. This was attained using a cross-platform application called "MARTApp" developed at Mistral [2] (paper in preparation). The first automatic steps performed by the application for each polarization, are: (i) conversion of the raw data to nexus format (.hdf5), (ii) normalization of each image using the corresponding FF, (iii) alignment and average of images. Different alignment methods are available to guarantee a sub-pixel quality of the alignment. The output of these 3 steps are 2 transmission images, 1 for each polarization (T_{C^+}, T_{C^-}). A correction factor is applied on one of them to assure that the average intensity in the field of view is the same for the 2 polarization. This is required to compensate for FF instabilities that may appear just after the polarization switch. Finally, these 2 images are aligned and the following formula is used to obtain the X-ray magnetic circular dichroism (XMCD) signal:

$$\text{XMCD signal} = \frac{-\log(T_{C^+}) - (-\log(T_{C^-}))}{2} \tag{1}$$

This signal depends on the magnetization component on the X-ray direction, which is, for sample tilt 0°, the m_z component and it is the one reported in Figure 2l and Figure 3a–d of the main text.

It is worth to note that the contrast in the obtained XMCD images is determined by the projection of the magnetic moment along the direction of the X-ray beam: red color indicates the local magnetic moment parallel to the X-ray beam, whereas blue color is antiparallel.

II. DETAILS ON ELECTRON HOLOGRAPHY

Electron holograms of of permalloy nanoflowers were recorded using a double-corrected FEI Titan^3 80-300 transmission electron microscope (ThermoFisher Comp., USA) operated at an acceleration voltage of 300 kV in magnetic field-free Lorentz mode (objective lens switched off). To extend the holographic field-of-view to 2 μm, i.e., by a factor 3 larger with respect to the conventional Lorentz mode, the transfer lenses within the imaging corrector were changed yielding the desired demagnification. Using this imaging mode, a fringe spacing of 8 nm at a fringe contrast of 10% was achieved in the electron holograms, which were acquired by a 4k $\times$ 4k Gatan Oneview CMOS camera. The amplitude and phase image reconstructions, the separation of electric and magnetic phase shifts, as well as the calculation of the final projected magnetic induction map (in-plane components) from the phase images were performed as described by Volkov et al. [3].

III. DETAILS ON MAGNETO-OPTICAL KERR EFFECT (MOKE) MEASUREMENTS

A digitally enhanced wide-field Kerr microscope, operating based on MOKE, was employed to investigate the magnetic properties and hysteresis behaviour of arrays of 3D magnetic objects. The microscope was configured for a longitudinal setup with pure in-plane sensitivity, using blue light with a wavelength of 457 nm generated by light-emitting diodes [4, 5]. Magnetic hysteresis loops were measured by sweeping an external magnetic field H along the direction of the microscope's sensitivity axis and plotting the average grey level of a selected region of interest as a function of H, thereby capturing the magnetization component M parallel to H. Additionally, Kerr microscopy was used to image the evolution of magnetic domains during the magnetization reversal process. To ensure high-quality domain images, the image intensity was averaged over 16 image frames to reduce noise, and a reference image of a saturated state was subtracted from the live view. A piezo stabilization was also utilized to prevent image drift during the measurement process.

IV. MICROMAGNETIC SIMULATIONS

We investigate equilibrium magnetic states in an individual nanoflower. The dimensions of the simulated geometries are taken as in the experiment (Supporting Figs. 3, 4). The numerical analysis is performed using a simulation software MAGPAR [6, 7]. We account only for the exchange and magnetostatic interactions in magnetic energy. The micromagnetic parameters are as follows: the exchange stiffness $A = 13\,\mathrm{pJ/m}$ and the saturation magnetization $M_s = 796\,\mathrm{kA/m}$. The average tetrahedron size in the finite element mesh is 3 nm.

Any vector field, which is associated with a real system like a ferromagnetic object with its specific magnetization distribution can be characterized by different homotopy groups. For truly topologically trivial states, each and every of their topological charges will be trivial (i.e., strictly zero). However, the presence of a topologically nontrivial state renders one or several topological charges to be a nonzero integer number, while other charges could take any value. The dimensions of nanoflowers support meron magnetic states (vortices or antivortices). The topological charge relevant for merons in easy-plane magnets is given by the homotopy group $\pi_1(\mathbb{S}_1)$ and its topological charge (vorticity) q with $q = \pm 1$ for vortices and antivortices, respectively [8, 9]. This assures their topological stability. Indeed, the meron core, represented by a Bloch line in a 3D magnetization distribution, corresponds to a singular point in any 2D cut hosting circulation of the magnetization, which cannot be lifted by any continuous transformations of the magnetization field in the absence of sample boundaries. The Pontryagin index (the topological charge for $\pi_2(\mathbb{S}_2)$ homotopy group) is half-integer and the Hopf index (the topological charge for $\pi_3(\mathbb{S}_2)$ homotopy group) is zero for these textures.

To determine the equilibrium lowest-energy states, we rely on the so-called multistart approach of running the energy minimization from several initial states including random states, uniform states with magnetization along $\pm\hat{\boldsymbol{x}}$, $\pm\hat{\boldsymbol{y}}$, $\pm\hat{\boldsymbol{z}}$ (here, $\hat{\boldsymbol{z}}$ is aligned with the symmetry axis C_4 of the geometry; $\hat{\boldsymbol{x}}$ points along the direction d_1, see Supporting Fig. 3), vortices and antivortices of different polarities and circulations placed in the center or shifted from the geometric center of the geometry. For vortices, we also tested different combinations of directions of magnetization within the petals.

The lowest-energy state depends on the thickness of the magnetic object (Supporting Fig. 11). For thicknesses below 50 nm, it corresponds to the magnetic flower state with the mirror plane parallel to the shortest diameter of the sample (i.e., along the direction d_1 shown in Supporting Fig. 3), see Supporting Fig. 11(a–f). To localize topologically nontrivial magnetization states, we calculate the distribution of the topological charge flux density [10, 11]. Its Cartesian components read

$$\Omega_l = \frac{1}{8\pi}\varepsilon_{lno}\varepsilon_{ijk}m_i\partial_n m_j\partial_o m_k, \quad i,j,k,l,n,o = x,y,z, \tag{2}$$

where ε_{ijk} is the Levi–Civita tensor and $\boldsymbol{m}(\boldsymbol{r})$ is the unit vector of magnetization. This approach has been proven a convenient tool for the analysis of magnetic states in complex-shaped 3D nanoobjects [3, 12]. The detection of topologically nontrivial states in the magnetization field $\boldsymbol{m}$ can be done by tracking maxima of the normalized topological charge values $\tilde{\Omega} = \Omega/|\Omega_{\max}|$, where $\Omega = |\boldsymbol{\Omega}|$. Magnetic nanoflower objects accommodate also edge states, which are shown by isosurfaces $\tilde{\Omega} = 0.19$ in Supporting Fig. 11(c,f).

The lowest-energy state of a 50-nm-thick nanoflower is a vortex. It can be obtained in several ways: (i) starting simulations from the uniform state pointing upward, $\boldsymbol{m} = \hat{\boldsymbol{z}}$, and (ii) by solving the Landau–Lifshitz–Gilbert equation for 10 ns in MagPar starting from the vortex state in the center and possessing a Bloch line of the width which is 3 times larger than the equilibrium width. This magnetic state is characterized by its polarity $p = \pm 1$ (i.e., the direction of magnetization in the Bloch line, pointing upward or downward) and circulation of magnetization $C = \pm 1$ (counter-clockwise or clockwise) in the vicinity of its Bloch line. Together, polarity and vorticity give the Pontryagin index $Q_P = pq/2$ [13]. The vortex is shifted from the geometric center of the nanoflower along one of the symmetry planes between petals (Supporting Fig. 11(g–i)). Any observed small shifts of the final position of vortices from the symmetry plane are within the precision of the energy minimization algorithm. Because of a complex shape of the boundary of a nanoflower object, the vortex state is supplemented by four edge states (Supporting Fig. 11(g) and isosurfaces $\tilde{\Omega} = 0.04$ in Supporting Fig. 11(i)).

The petals of nanoflowers are almost uniformly magnetized. Within the numerical accuracy, the energy of the state does not depend on the mutual orientation of the magnetization in petals, circulation and polarization of the vortex. This allows us to conclude that the observed asymmetric ground state is determined not by the effects of geometric curvature stemming from the exchange interaction. This expectation is supported by the established absence of any coupling between the vortex polarity p and convexity of the shell [14]. Furthermore, the observed asymmetric ground state is not a result of the nonlocal chiral symmetry break, which is also dependent on the convexity of the shape [15]. Therefore, the formation of the shifted vortex state can be qualitatively explained as follows. For each petal, the energetically favorable magnetization direction lies in the vertical symmetry plane of the petal because of its narrow

elongated shape. The value of the magnetostatic energy can be determined by the balance of the surface and volume magnetostatic charges, $\sigma(\boldsymbol{r}) = \boldsymbol{m}\cdot\boldsymbol{\nu}$ with $\boldsymbol{\nu}$ being the surface normal, and $\lambda(\boldsymbol{r}) = -\nabla\cdot\boldsymbol{m}$, respectively:

$$E_{\text{ms}} = \frac{M_s^2}{2}\iint dSdS'\frac{\sigma(\boldsymbol{r})\sigma(\boldsymbol{r}')}{|\boldsymbol{r}-\boldsymbol{r}'|} + \frac{M_s^2}{2}\iint d\boldsymbol{r}d\boldsymbol{r}'\frac{\lambda(\boldsymbol{r})\lambda(\boldsymbol{r}')}{|\boldsymbol{r}-\boldsymbol{r}'|} + M_s^2\iint d\boldsymbol{r}dS'\frac{\lambda(\boldsymbol{r})\sigma(\boldsymbol{r}')}{|\boldsymbol{r}-\boldsymbol{r}'|}. \tag{3}$$

Here, the first two terms are responsible for the pairwise interaction between the magnetostatic charges and the last term shows their cross-interaction. The first two terms in Eq. (3) are quadratic by their charges and result in the reduction of the absolute values of σ and λ. The last term is bilinear by the volume and surface charges. This term can result in a stabilization of chiral magnetic microstructures in nanoobjects possessing low geometric symmetry due to balancing of the contributions from σ and λ [3]. Supporting Figs. 16(a–c) and (d–f) show the distribution of the surface and volume magnetostatic charges for shifted and centered vortices, respectively. We note that in all cases $\lambda > 0$ appears in the vicinity of $\sigma < 0$ and vice versa. However, for a shifted vortex, the surface and volume charges of opposite sign are located closer together near the side face of a nanoflower. This closer distance enhances their interaction strength and leads to lowering of the value of the cross-interaction term in Eq. (3) in comparison with a more symmetric case.

The vortex energy has a logarithmic divergence with the system radius R, $E \propto \ln(R/w)$, where w is the Bloch line width [13, 16]. In the exchange approximation, the energy of the system increases with the growth of R and decreases with the growth of w. However, in anisotropic systems, the value of w is mainly determined by the competition between exchange and anisotropic interactions. The dependency of w on the system size R is exponentially weak [17], while the magnetostatics can introduce the spatial inhomogeneity in w [18]. The metastability of such vortex solution in the absence of magnetostatics is well established in discrete systems [13].

We note that the competition between the exchange interaction favoring the uniform magnetization distribution and magnetostatics minimizing the self- and cross-interaction of magnetistatic charges is the main reason for the formation of the meron magnetic states in the samples with the complex shape of their boundary.

To estimate the shape of a Bloch line for a vortex shifted along the symmetry plane between petals, we performed ten simulations of the same magnetization distribution using different meshes of the same average tetrahedron size. In each mesh vertex, the value of $\boldsymbol{\Omega}$ is calculated. The combined distribution of $\boldsymbol{\Omega}(\boldsymbol{r})$ from ten simulations allows us to effectively increase the spatial resolution in the determination of the topological charge flux density. We determine the average direction of the Bloch line and build 32 slices containing 507 to 1267 combined mesh vertices with $\tilde{\Omega} > 0.1$. Within each slice, the center of mass of $\tilde{\boldsymbol{\Omega}}$ is calculated as the estimate of the Bloch line position $\boldsymbol{\gamma}$ in the given slice (Supporting Fig. 9(a)). With $\boldsymbol{\gamma}$, we calculate the discrete tangential, normal and binormal vectors according to [19] (Supporting Fig. 9(b–d)). We note that only the tangential vector is well-defined, which indicates that the Bloch line is represented by a plane curve $\boldsymbol{\gamma}$, whose curvature is about $0.027\,\text{nm}^{-1}$, see Supporting Figs. 9 and 10.

Several examples of excited states are shown in Supporting Fig. 12. A magnetic flower state has higher energy than a shifted vortex and it is followed by vortices with different configuration of magnetization within the petals. The most symmetric configurations like vortex with the magnetization parallel to the side faces of the sample (this state is obtained starting from the centered vortex possessing a Bloch line of the width as in equilibrium) as well as antivortex have the highest energy. The stability of the antivortex state is in agreement with previous reports considering planar geometries with four-fold symmetry [20, 21]. We note that the Gaussian curvature of the nanoflowers is positive. This contributes to the preference of stabilization of a vortex instead of antivortex as seen in our simulations (Supporting Fig. 12(d–f)). This finding is in agreement with previous studies of meron structures in flexible disks [22]

The results presented in this work can be correlated with the genus 2 of the geometry of an individual nanoflower [23] if the entire surface of the nanoflower is considered. The individual nanoflower hosts eight edge states (four of them are visible at the top side; they are marked as "ES" in Fig. 3(f) of the main text and Supporting Fig. 11(g)). The edge states are half-antivortices with the total vorticity $q_{\text{av}} = (-1/2)\times 8 = -4$. Taking into account surface vortices at the petals ends, the total vorticity of the vortices in the sample is $q_{\text{v}} = 2+4 = 6$. Thus, the total vorticity of all topological magnetic structures at the surface of the nanoflower is $q_{\text{tot}} = q_{\text{v}} + q_{\text{av}} = +2$ as it should be for an object with the geometry homeomorphic to a sphere.

Supporting Fig. 13 shows changes of the vortex state depending on the magnetization configuration within the petals. These states are obtained starting from the centered vortex with possessing a Bloch line of the width as in equilibrium defined within the d_1 region (see Supporting Fig. 3) and magnetization pointing inward or outward along the petals. The lowest energy corresponds to the vortex shifted from the geometric center along the symmetry plane of the geometry (Supporting Fig. 13(a)). In this case, the petals have their magnetization direction pairwise inward and outward from the center of the object. The state with only one petal having opposite magnetization with respect to other three petals has higher energy (Supporting Fig. 13(b)). In this case, the vortex lies on the line connecting petals with opposite magnetization. Other two high-energy states (Supporting Fig. 13(c,d)) correspond to symmetric configurations with vortices located in the geometric center of the sample.

Supporting Fig. 14 shows two magnetic flower states, which are similar to those observed experimentally (compare top and bottom row). The magnetic flower state co-aligned with the direction d_1 (see Supporting Fig. 3) has lower energy.

Having experimental evidence that an individual nanoflower object can host several Bloch lines (see Figure 3c, main text), we analyze a possibility to relax a similar state in simulations. Starting from different combinations of pairs of vortices and antivortices, we can stabilize only a pair of vortices of opposite circulations, see Figure 3j, main text. The simulated state resembles the experimentally observed state (see Figure 3c, main text) with both Bloch lines magnetized along the line connecting opposite petals.

Supporting Fig. 17 shows in-plane hysteresis loops for an individual nanoflower geometry. The remanent states are the magnetic flower states, c.f. Supporting Fig. 14(a,c).

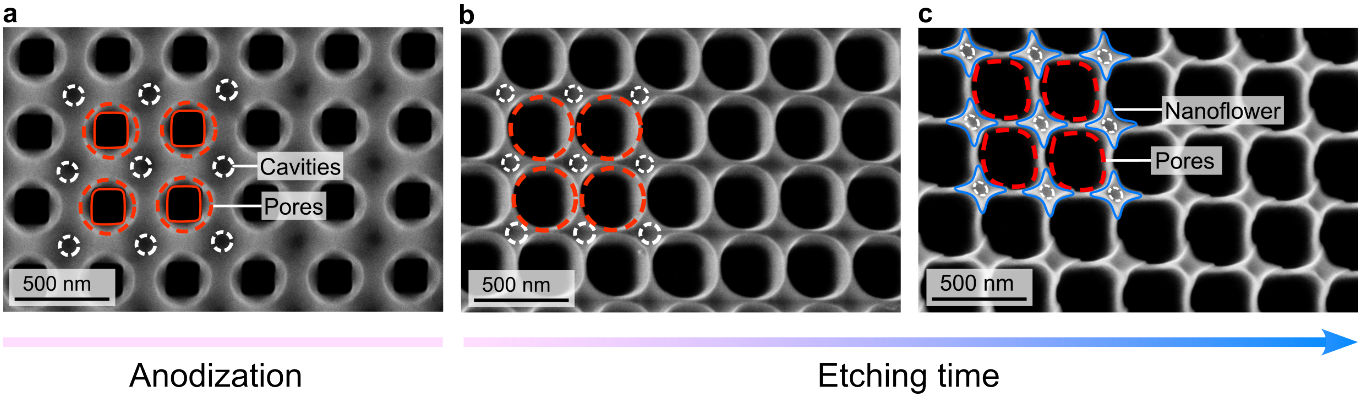

Supporting Fig. 1. Sample fabrication. After imprinting a polished aluminum foil with a nickel stamp, an array of nanopores is formed. A subsequent anodization step increases the depth of nanopores and forms additional cavities between them (a). The anodization process allows to tune the depth of nanopores depending on the duration of the anodization process. The following wet etching step widens the pores and cavities, so that the cavities start to form an array of nanoflower-like geometries, connected by nanopores (valleys). Here, the sample was etched in a controlled way for (b) 6 min and (c) 7.5 min to achieve desirable length of petals of nanoflowers (around 40 nm between the flower petals).

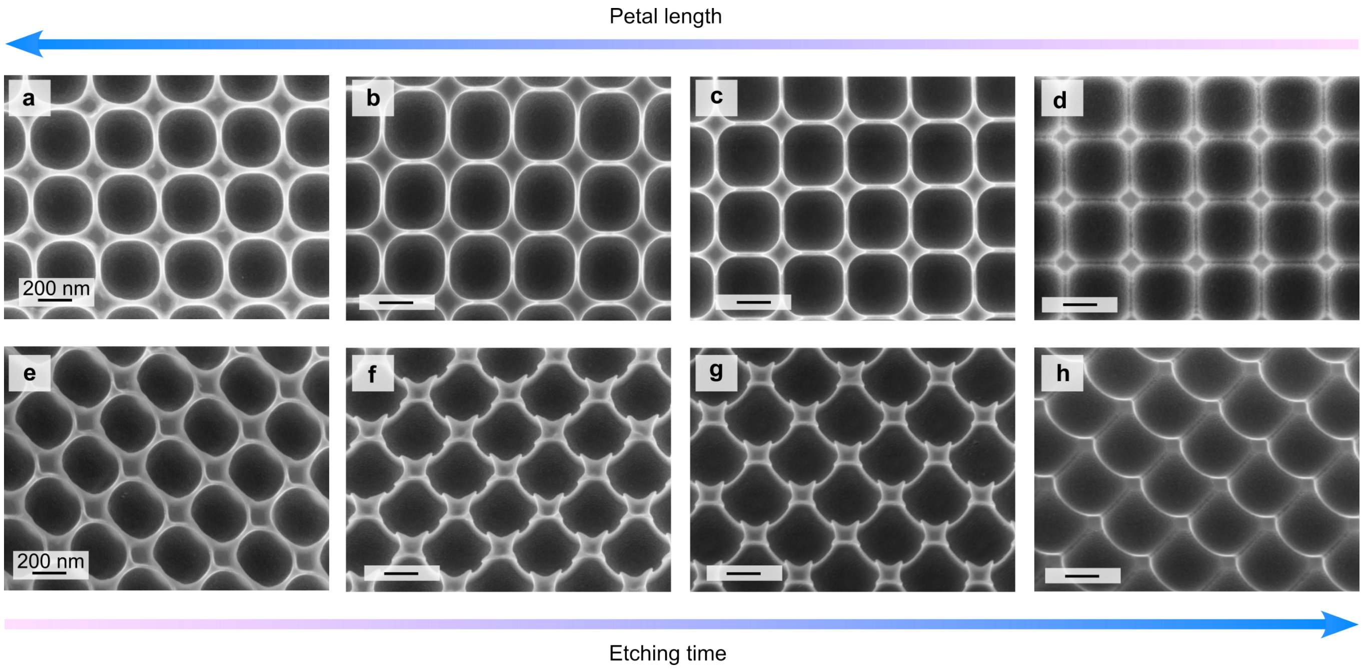

Supporting Fig. 2. Tailoring 3D nanoscale geometry. Top row (a-d) presents top-view SEM images of 3D nanostructures. Bottom row (e-h) displays the respective tilted-view SEM images taken at 30° tilt angle. The petal length of nanoflowers is finely adjusted through a controlled wet etching process. As the etching duration increases, the petal length decreases, allowing for precise tailoring of the nanostructure geometry. In this manuscript, the geometry shown in images (c, g) was selected for the detailed analysis.

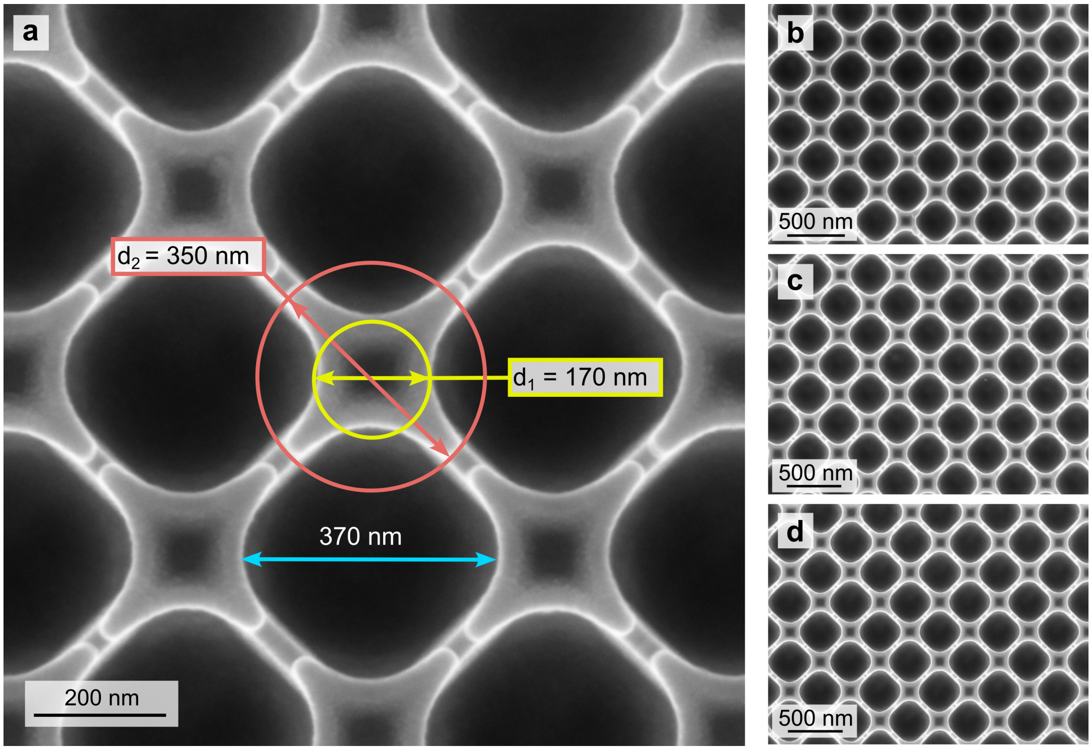

Supporting Fig. 3. Dimensions of the studied sample geometry. (a) A top-view SEM image shows a nanoflower structure after deposition of a 50-nm-thick permalloy thin film. Relevant dimensions of the studied sample are indicated: inner diameter of the nanoflower is equal to 170 nm, diagonal is 350 nm. The diameter of valleys (distance between flowers), which connect nanoflowers, is 370 nm. The distance between the petals of nanoflowers is around 40 nm. (b–d) Top-view SEM images taken from different regions of the sample (distance between regions is about 100 μm). The geometric parameters are given in Table I.

Panel	d_1, nm	d_2, nm	Distance between flowers, nm	Distance between petals, nm
b	169 ± 5	346 ± 12	368 ± 7	41 ± 11
c	169 ± 4	348 ± 12	371 ± 6	40 ± 14
d	168 ± 5	351 ± 12	370 ± 6	42 ± 15

Supporting Table I. Geometric parameters of nanoflowers measured at different locations of the sample shown in Supporting Fig. 3(b–d).

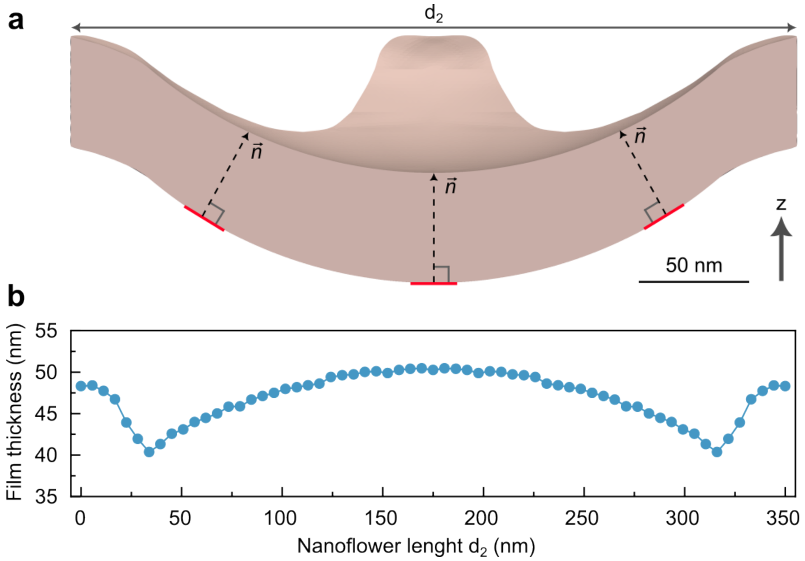

Supporting Fig. 4. (a) A cross-section of the finite element model of a nanoflower geometry used in micromagnetic simulations. The geometry is based on the scanning electron microscopy image shown in Supporting Fig. 3. The image shows variation of the film thickness over the nanoflower geometry. (b) Plot of the film thickness along the direction, which is normal to the surface of the flower geometry.

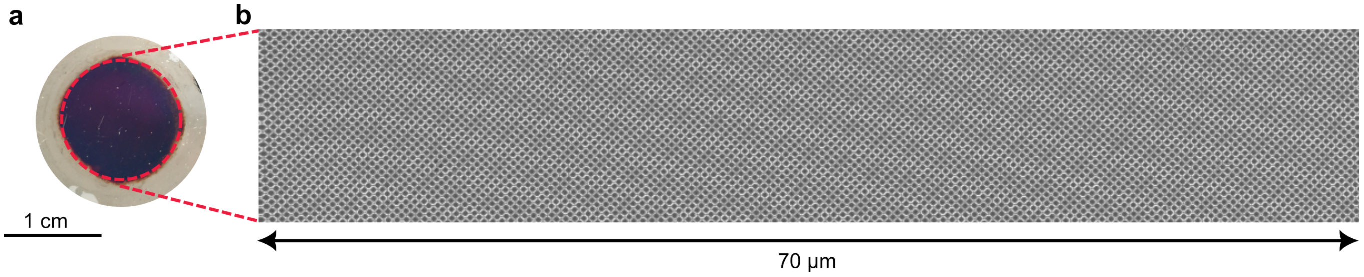

Supporting Fig. 5. Scalability of the method to prepare 3D nanostructures. Panel (a) shows an optical photograph of the fabricated sample. A nanoflower array is located within the area marked with a red circle. For this manuscript, a sample with nanoflowers within the area with diameter of 12 mm was prepared. (b) SEM image shows a large-scale top-view image of nanoflowers, containing no defects over 70 μm length.

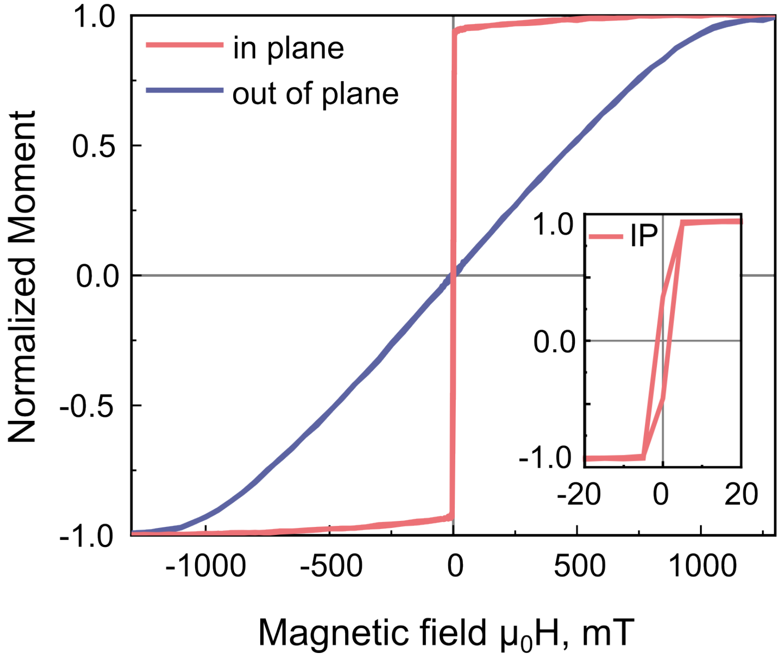

Supporting Fig. 6. Magnetic hysteresis loops of a reference 30-nm-thick permalloy thin film, sputtered on a planar SiOx substrate. The blue loop represents the measurement in an out-of-plane magnetic field, i.e. field is applied perpendicular to the sample surface. The red loop shows the measurement in an in-plane magnetic field, i.e. field is applied parallel to the sample surface. The inset shows a close-up of the in-plane hysteresis loop. The hysteresis loops were obtained at room temperature using a commercial MicroSense EZ7 vector Vibrating Sample Magnetometer (VSM). Magnetic field of 1.8 T was applied to saturate the sample. Saturation magnetization (M_S) was calculated to be 756 kA/m. This value was used for micromagnetic simulations.

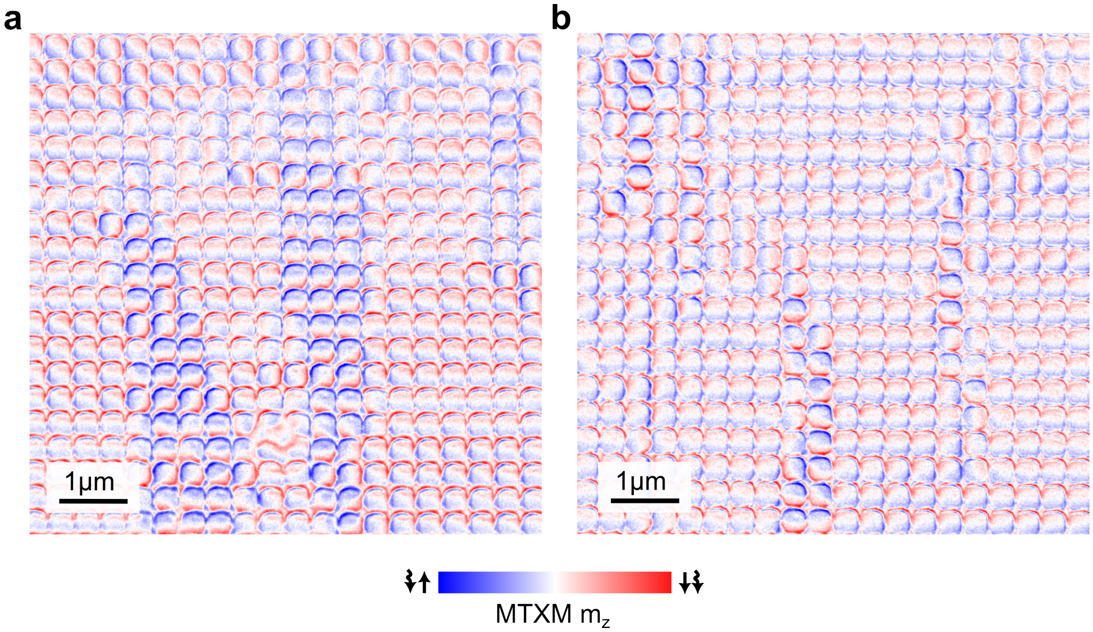

Supporting Fig. 7. MTXM images taken at different locations of the nanoflower sample capped with 50-nm-thick permalloy film. Images (a, b) were taken under same conditions as described in the experimental section of the main text. These magnetic patters (a, b) are similar to those shown in main text, Figure 2l.

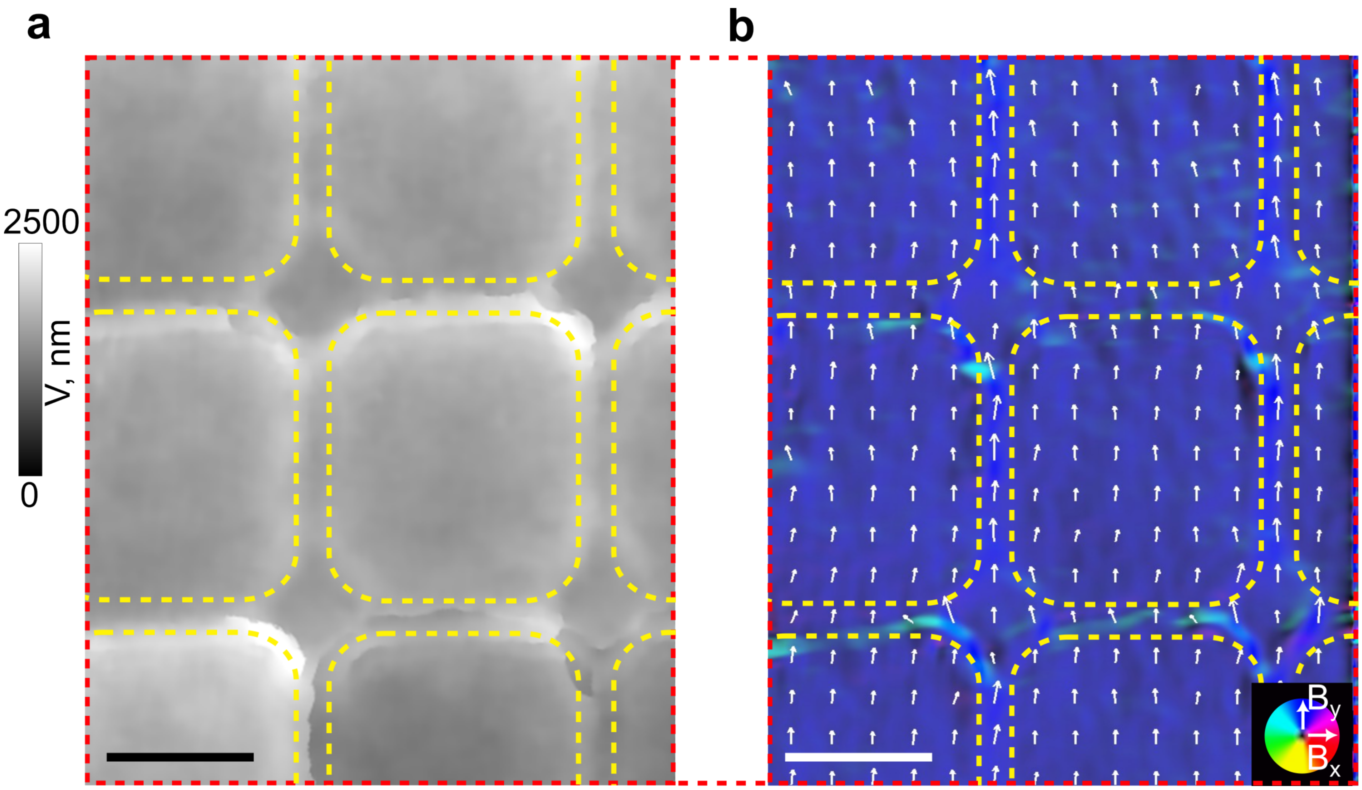

Supporting Fig. 8. Transmission electron microscopy (TEM) off-axis electron holography of permalloy nanoflower array. (a) Reconstructed electrical phase shift showing the (projected) morphology of the structure. (b) Magnetic projected induction map (B-field) obtained from the reconstructed magnetic phase shift at the region of the red-dashed box indicated in (a). The yellow boxes indicate the valleys of the permalloy nanoflower array. The scale bar is 200 nm.

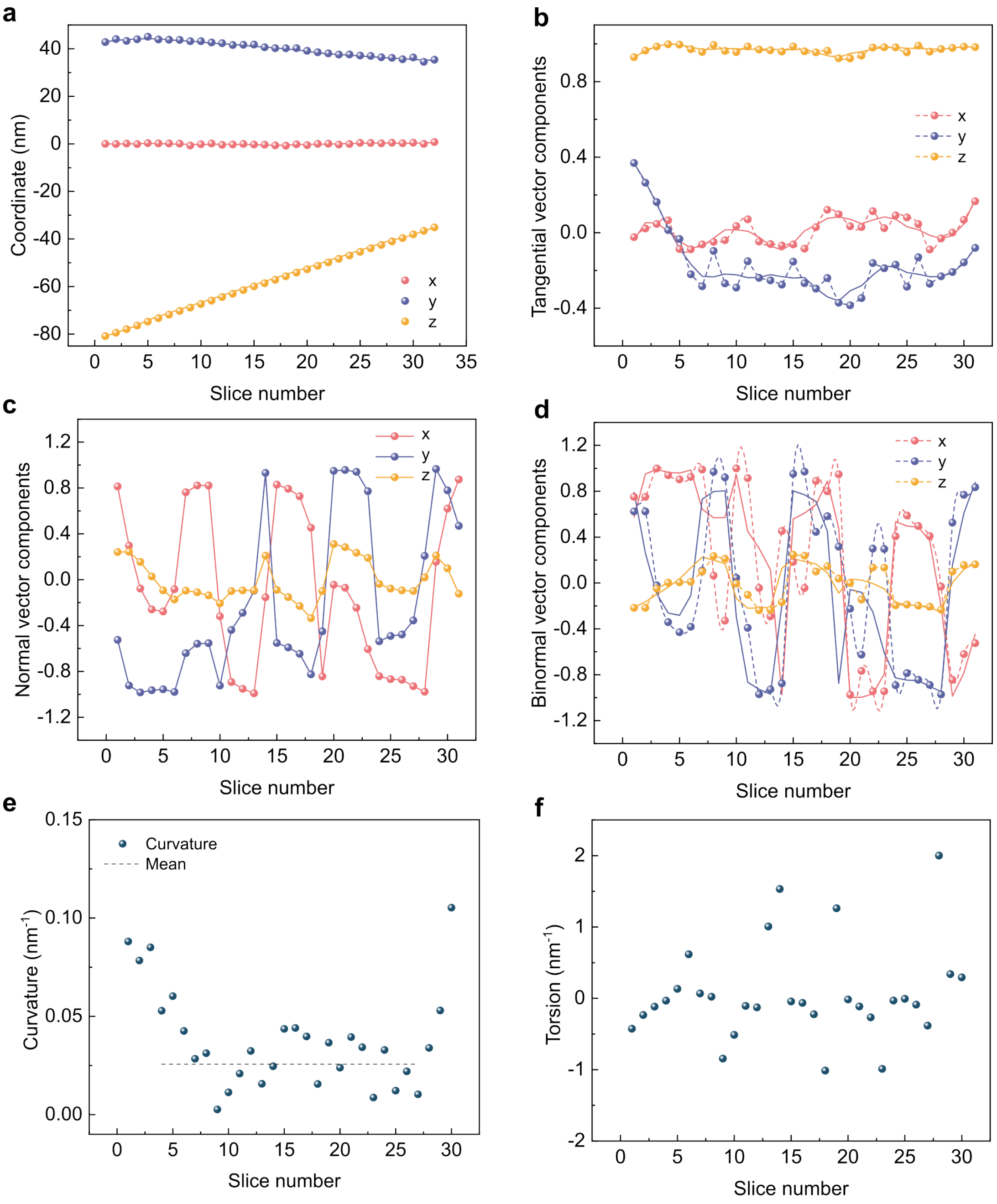

Supporting Fig. 9. A profile of the Bloch line for a vortex state with the polarity up (i.e, $p = +1$). The profile is determined from the analysis of a stack of slices of the sample in simulations. (a) Cartesian coordinates of the Bloch line. (b) Cartesian components of the tangential, (c) normal and (d) binormal vectors along the Bloch line. In (b) symbols correspond to the raw data obtained from (a) and solid lines represent the vector components after smoothing to reduce noise. The distribution of (e) curvature and (f) torsion along the Bloch line. The black curve shows the mean value of curvature, $\langle\kappa\rangle = 0.026\,\mathrm{nm}^{-1}$

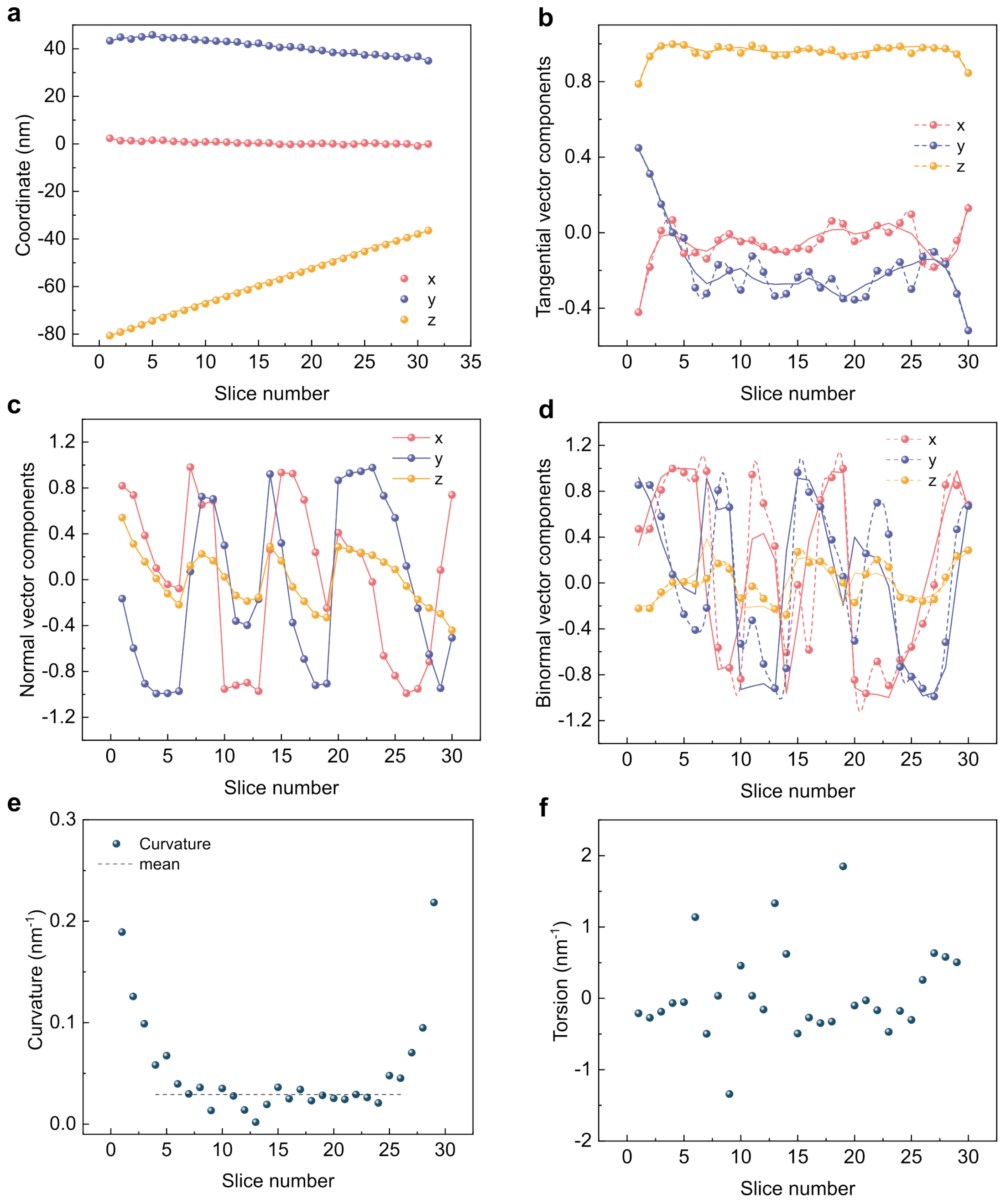

Supporting Fig. 10. A profile of the Bloch line for a vortex state with the polarity down (i.e, $p = -1$). The profile is determined from the analysis of a stack of slices of the sample in simulations. (a) Cartesian coordinates of the Bloch line. (b) Cartesian components of the tangential, (c) normal and (d) binormal vectors along the Bloch line. In (b) symbols correspond to the raw data obtained from (a) and solid lines represent the vector components after smoothing to reduce noise. The distribution of (e) curvature and (f) torsion along the Bloch line. The black curve shows the mean value of curvature, $\langle\kappa\rangle = 0.029\,\mathrm{nm}^{-1}$

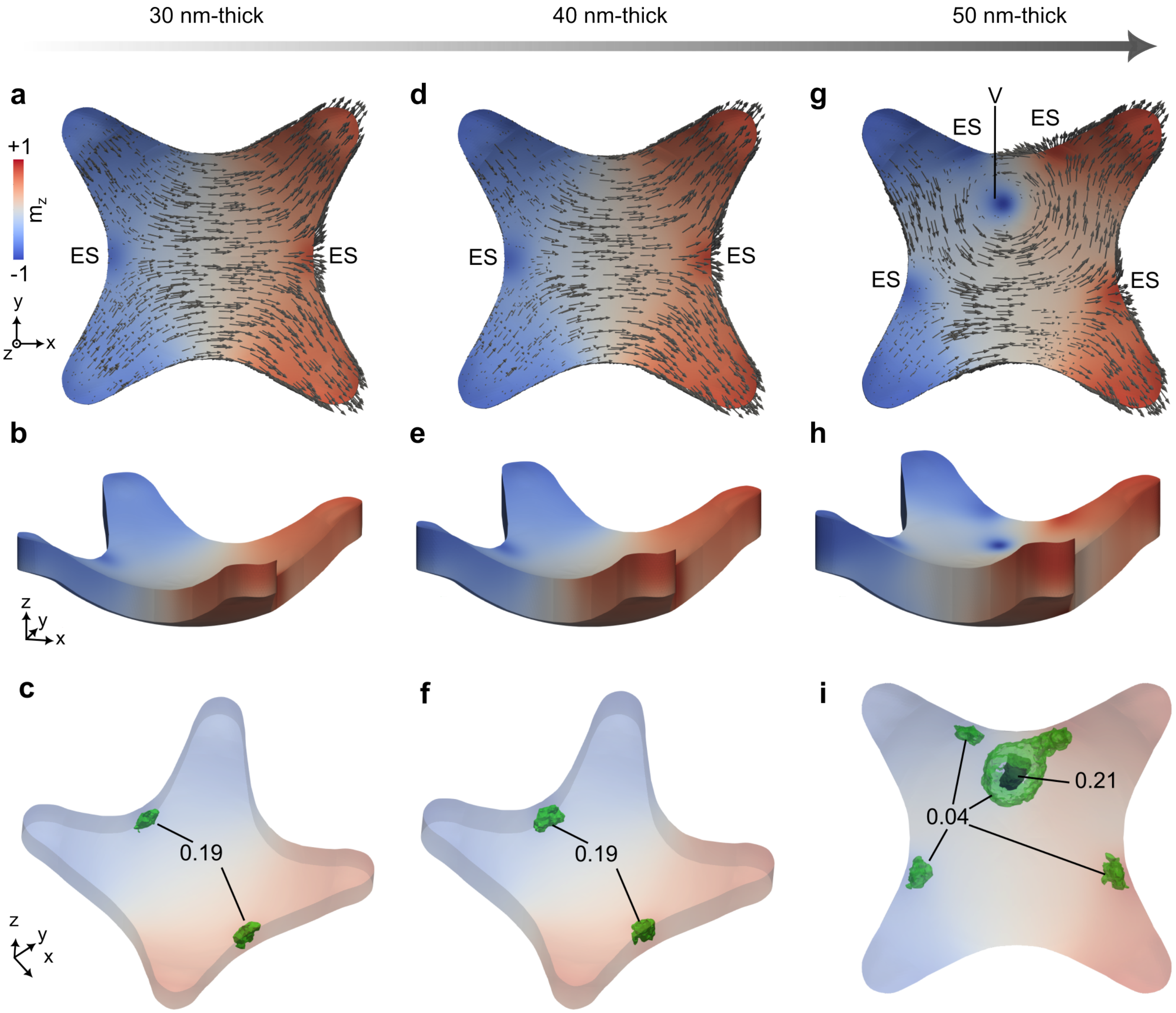

Supporting Fig. 11. Simulated ground energy states for nanoflowers of different thicknesses. For (a–c) 30-nm-thick and (d–f) 40-nm-thick structures, the lowest energy state is a magnetic flower state. (g–i) For 50-nm-thick films, the ground state is the state with a shifted vortex. The topological charge densities $\tilde{\Omega}$ are shown in green color and equal to 0.19 for 30-nm-thick and 40-nm-thick flowers (indicating edge states). For a 50-nm-thick sample, $\tilde{\Omega} = 0.21$ (for the vortex structure) and $\tilde{\Omega} = 0.04$ (for edge states). In panels (a, d, g), "ES" stands for the edge state and "V" stands for the vortex state.

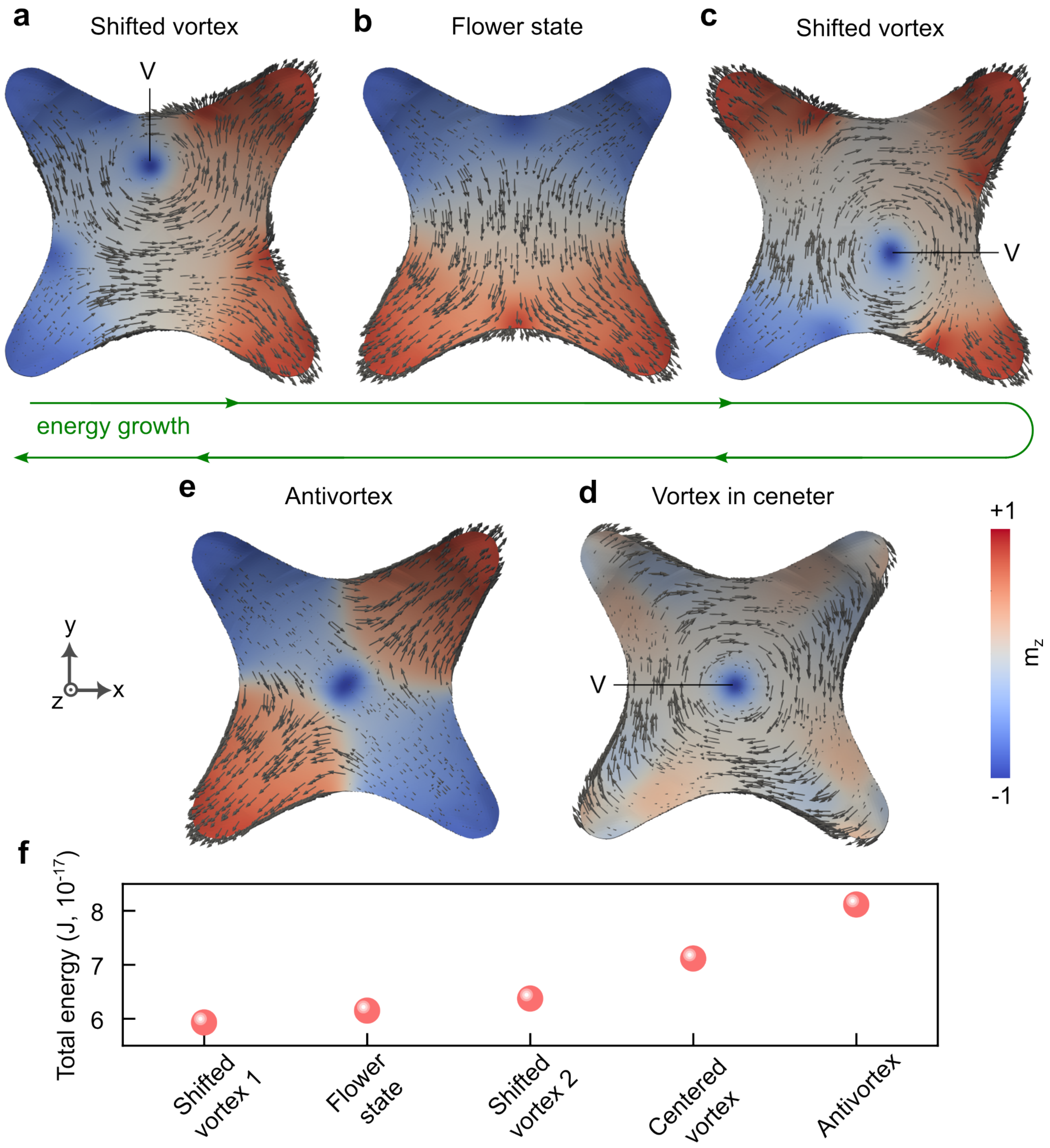

Supporting Fig. 12. Different simulated magnetic states in 50-nm-thick nanoflowers and the corresponding energy of these states ("V indicates vortex center"). (a) The lowest energy state is a shifted vortex, followed by (b) the magnetic flower state, (c) shifted vortex with asymmetric magnetization in petals, (d) vortex in the center of a nanoflower and (e) antivortex state. (f) Energies E_{tot} of the states shown in panels (a–e).

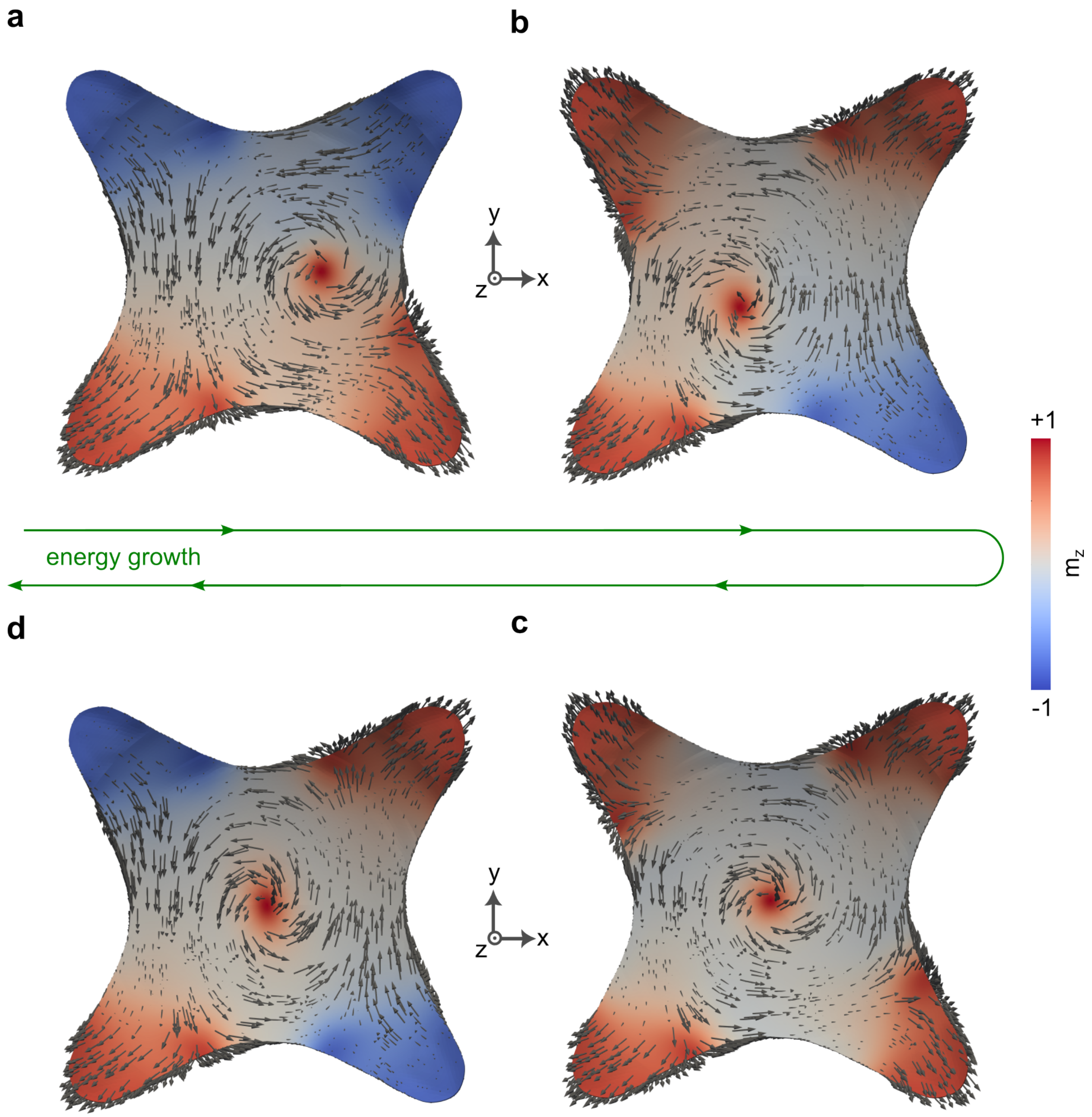

Supporting Fig. 13. Examples of vortices in 50-nm-thick nanoflowers arranged accordingly to the increase of their energy. (a) A vortex shifted from the geometric center of the object along the symmetry plane between petals. Pairs of petals are oppositely magnetized. (b) Vortex shifted from the geometric center of the object along the line connecting two opposite petals if one of the petals is magnetized oppositely to three others. (c, d) High-symmetry configurations with a vortex in the geometric center of the object.

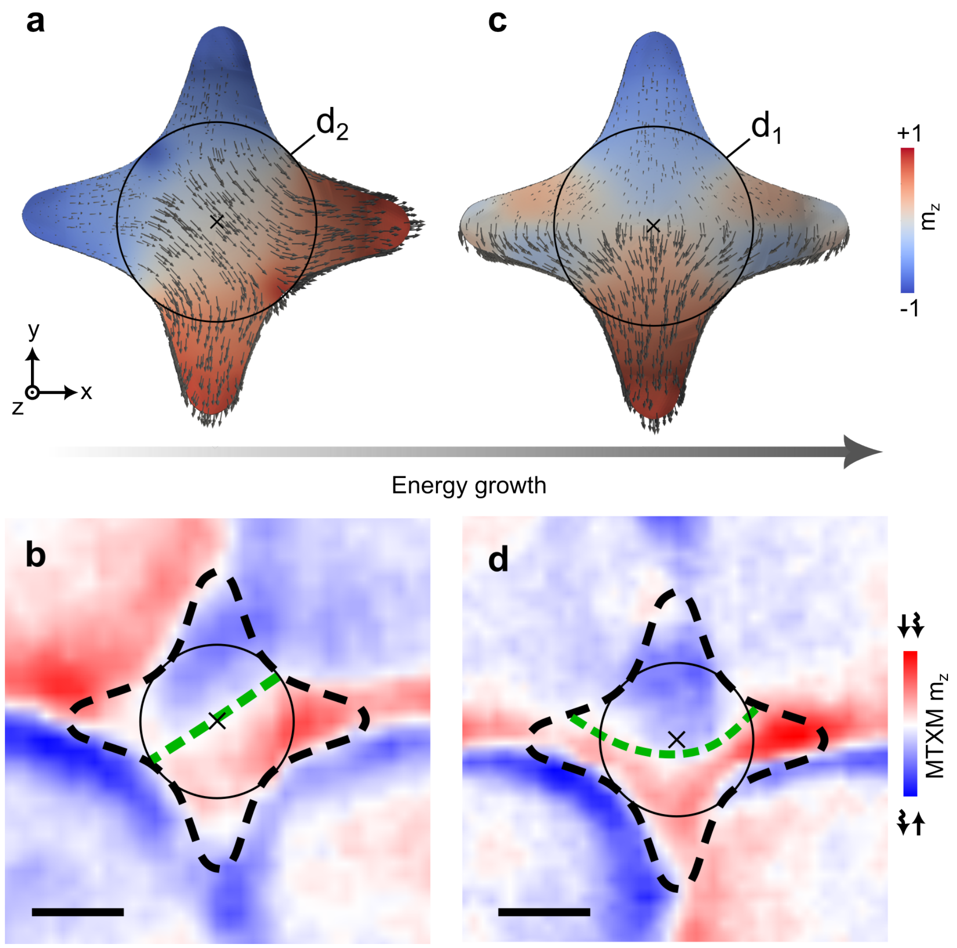

Supporting Fig. 14. Two configurations of the magnetic flower states, arranged accordingly to the increase of their energy. A magnetic flower state with the symmetry plane (a, b) between and (c, d) along the petals. The top row shows the simulated, while the bottom row displays the experimental states.

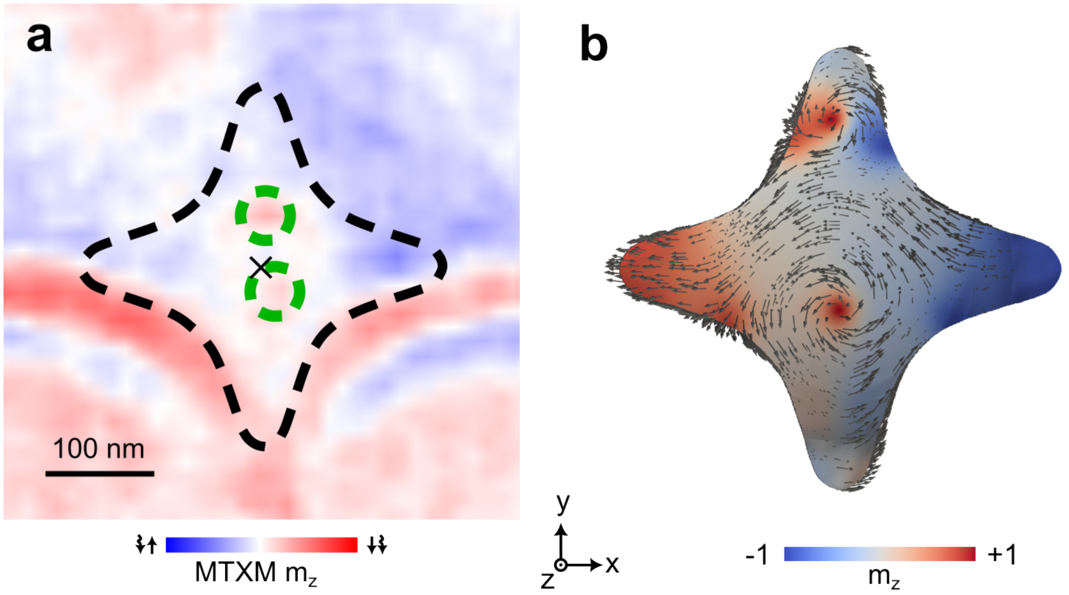

Supporting Fig. 15. An individual nanoflower with two Bloch lines. (a) Experimental MTXM image of the individual nanoflower hosting two Bloch lines. (b) Simulated state.

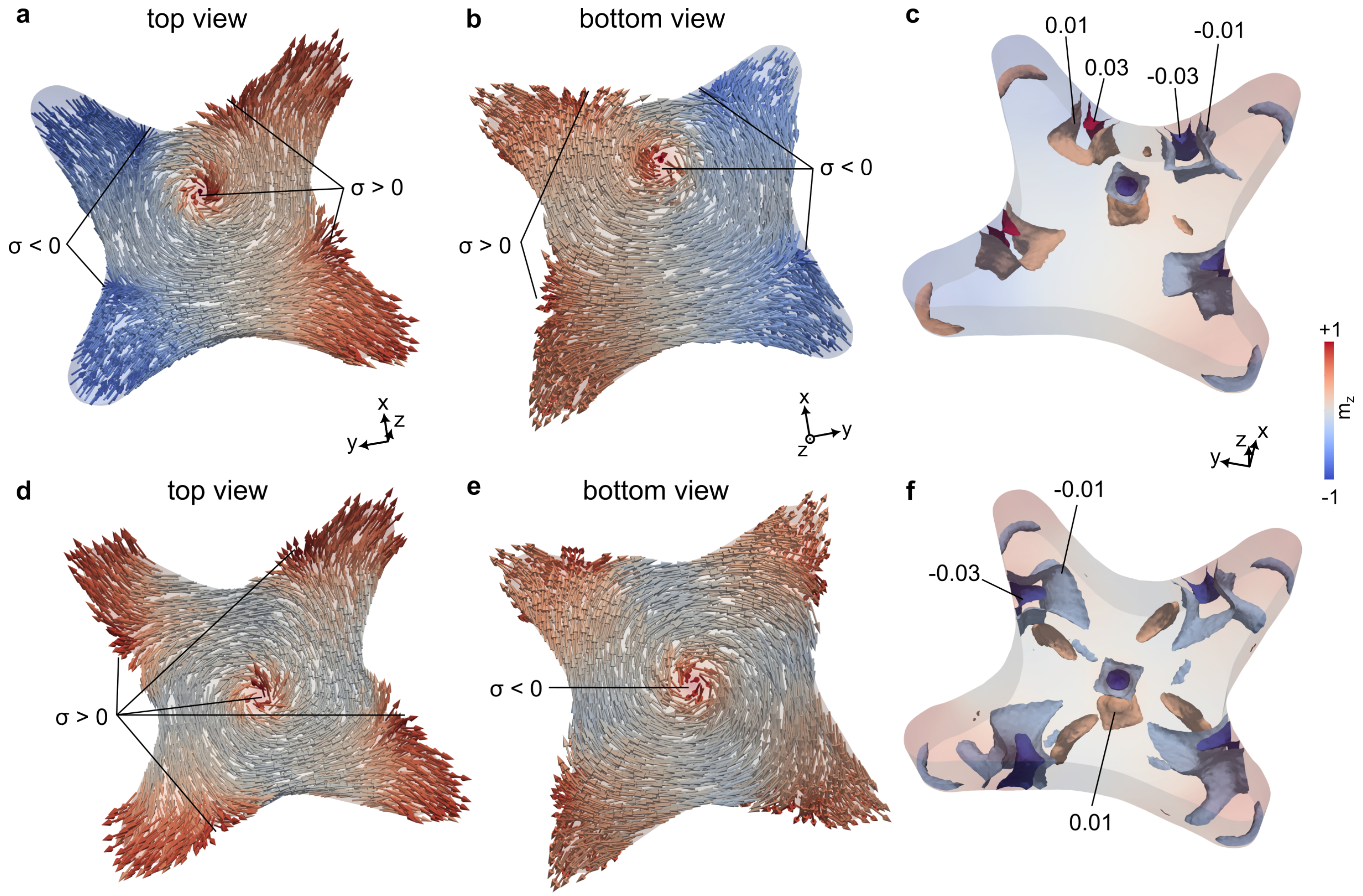

Supporting Fig. 16. Magnetostatic charges in a nanoflower. (a) Top- and (b) bottom-view of a magnetic nanoflower hosting a shifted vortex (ground state). Arrows indicate the magnetization direction. Visible regions with positive and negative surface magnetostatic charges σ are indicated. (c) Isosurfaces indicating the distribution of the volume magnetostatic charge $\lambda = \pm 0.01$ and $\lambda = \pm 0.03$ at the Bloch line and edge states, respectively. There are also small regions with a finite divergence of magnetization at the ends of the petals. (d–f) Same for the high-energy stable state with the magnetic vortex in the geometric center of the object.

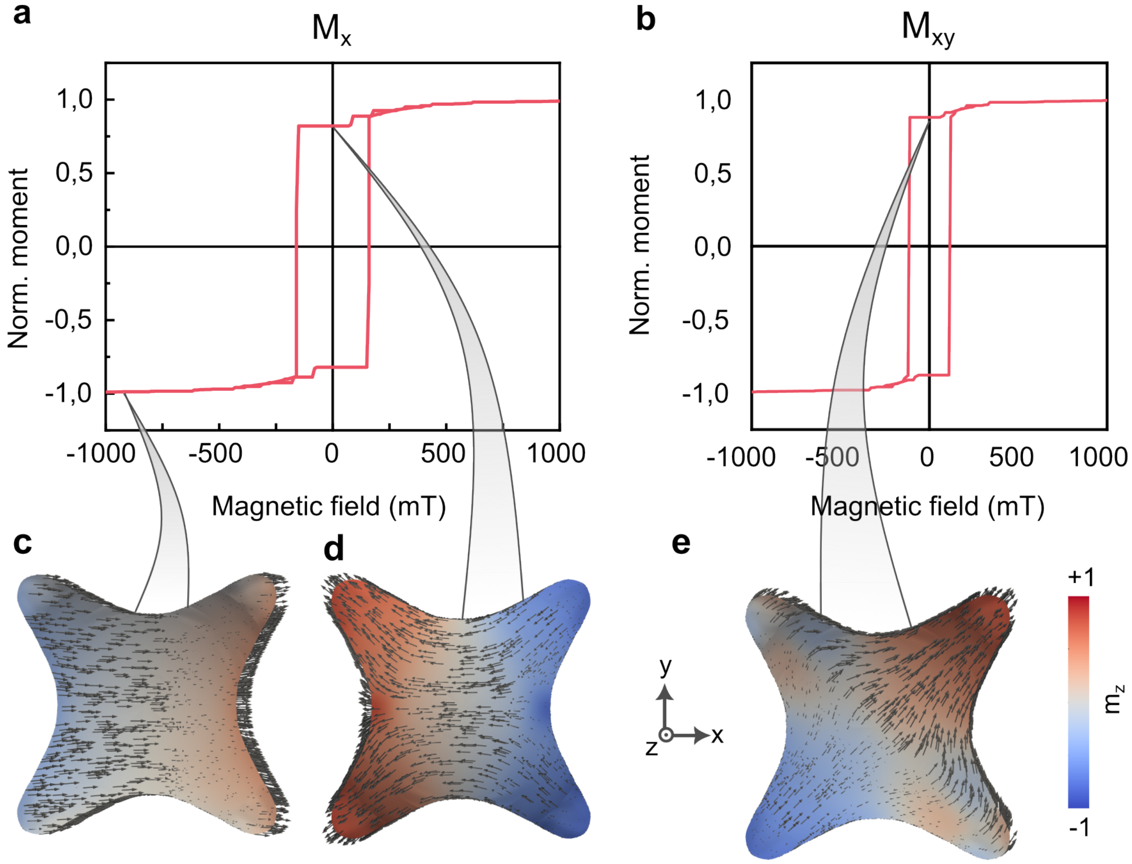

Supporting Fig. 17. Simulated in-plane hysteresis loops of an individual nanoflower. Magnetic field is applied along (a) $\hat{\boldsymbol{x}}$ and (b) $\hat{\boldsymbol{x}}+\hat{\boldsymbol{y}}$ directions. (c) Magnetic state close to saturation in the field oriented along the $\hat{\boldsymbol{x}}$ direction. Remanent states for the cases of fields applied along (d) $\hat{\boldsymbol{x}}$ and (e) $\hat{\boldsymbol{x}}+\hat{\boldsymbol{y}}$ directions.

REFERENCES

[1] A. Sorrentino, J. Nicolás, R. Valcárcel, F. J. Chichón, M. Rosanes, J. Avila, A. Tkachuk, J. Irwin, S. Ferrer, and E. Pereiro, Mistral: a transmission soft x-ray microscopy beamline for cryo nano-tomography of biological samples and magnetic domains imaging, Journal of synchrotron radiation **22**, 1112 (2015).

[2] Magnetic analysis & reconstruction of tomographies app (martapp), https://github.com/ALBA-Synchrotron/MARTApp, accessed 2024.11.18.

[3] O. M. Volkov, D. Wolf, O. V. Pylypovskyi, A. Kákay, D. D. Sheka, B. Büchner, J. Fassbender, A. Lubk, and D. Makarov, Chirality coupling in topological magnetic textures with multiple magnetochiral parameters, Nature Communications **14**, 1491 (2023).

[4] R. Schäfer and J. McCord, Magneto-optical microscopy, Magnetic measurement techniques for materials characterization , 171 (2021).

[5] I. Soldatov and R. Schäfer, Selective sensitivity in kerr microscopy, Review of Scientific Instruments **88** (2017).

[6] W. Scholz, J. Fidler, T. Schrefl, D. Suess, R. Dittrich, H. Forster, and V. Tsiantos, Scalable parallel micromagnetic solvers for magnetic nanostructures, Computational Materials Science **28**, 366 (2003).

[7] W. Scholz, magpar - parallel finite element micromagnetics package, http://www.magpar.net/, accessed 2024.11.18.

[8] N. D. Mermin, The topological theory of defects in ordered media, Reviews of Modern Physics **51**, 591 (1979).

[9] A. M. Kosevich, B. Ivanov, and A. S. Kovalev, Magnetic solitons, Physics Reports **194**, 117 (1990).

[10] N. Papanicolaou and T. Tomaras, Dynamics of magnetic vortices, Nuclear Physics B **360**, 425 (1991).

[11] N. Cooper, Propagating magnetic vortex rings in ferromagnets, Physical review letters **82**, 1554 (1999).

[12] C. Donnelly, K. L. Metlov, V. Scagnoli, M. Guizar-Sicairos, M. Holler, N. S. Bingham, J. Raabe, L. J. Heyderman, N. R. Cooper, and S. Gliga, Experimental observation of vortex rings in a bulk magnet, Nature Physics **17**, 316 (2021).

[13] F. G. Mertens and A. R. Bishop, Dynamics of vortices in two–dimensional magnets, in *Nonlinear Science at the Dawn of the 21th Century*, edited by P. L. Christiansen, M. P. Soerensen, and A. C. Scott (Springer–Verlag, Berlin, 2000) pp. 137–170.

[14] K. V. Yershov, V. P. Kravchuk, D. D. Sheka, and Y. Gaididei, Controllable vortex chirality switching on spherical shells, Journal of Applied Physics **117** (2015).

[15] D. D. Sheka, O. V. Pylypovskyi, P. Landeros, Y. Gaididei, A. Kákay, and D. Makarov, Nonlocal chiral symmetry breaking in curvilinear magnetic shells, Communications Physics **3**, 128 (2020).

[16] V. Kravchuk and D. Sheka, Thin ferromagnetic nanodisk in transverse magnetic field, Physics of the Solid State **49**, 1923 (2007).

[17] V. P. Kravchuk, D. D. Sheka, and Y. B. Gaididei, Equilibrium magnetisation structures in ferromagnetic nanorings, Journal of Magnetism and Magnetic Materials **310**, 116 (2007).

[18] A. Hubert and R. Schäfer, *Magnetic domains: The analysis of magnetic microstructures* (Springer Berlin Heidelberg, Berlin, 2009).

[19] S. Hu, Y. Jiang, and A. J. Niemi, Energy functions for stringlike continuous curves, discrete chains, and space-filling one dimensional structures, Physical Review D—Particles, Fields, Gravitation, and Cosmology **87**, 105011 (2013).

[20] K. Shigeto, T. Okuno, K. Mibu, T. Shinjo, and T. Ono, Magnetic force microscopy observation of antivortex core with perpendicular magnetization in patterned thin film of permalloy, Applied Physics Letters **80**, 4190 (2002).

[21] S. Gliga, M. Yan, R. Hertel, and C. M. Schneider, Ultrafast dynamics of a magnetic antivortex: Micromagnetic simulations, Physical Review B—Condensed Matter and Materials Physics **77**, 060404 (2008).

[22] B. Miranda-Silva, P. H. Taveira, A. W. Teixeira, J. M. Fonseca, L. N. Rodrigues, R. G. Elías, A. Riveros, N. Vidal-Silva, and V. L. Carvalho-Santos, Manipulating the shape of flexible magnetic nanodisks with meronlike magnetic states, Physical Review B **105**, 104430 (2022).

[23] O. M. Volkov, O. V. Pylypovskyi, F. Porrati, F. Kronast, J. A. Fernandez-Roldan, A. Kákay, A. Kuprava, S. Barth, F. N. Rybakov, O. Eriksson, S. Lamb-Camarena, P. Makushko, M.-A. Mawass, S. Shakeel, O. V. Dobrovolskiy, M. Huth, and D. Makarov, Three-dimensional magnetic nanotextures with high-order vorticity in soft magnetic wireframes, Nature Communications **15**, 2193 (2024).
